# A study on the significance of serine hydroxymethyl transferase expression and its role in bladder cancer

**DOI:** 10.1038/s41598-024-58618-2

**Published:** 2024-04-09

**Authors:** Si-Wei Su, Xian Chen, Guang Wang, Pei Li, Tong-Xin Yang, Ke-Wei Fang, Jing Wu, Jiong-Ming Li

**Affiliations:** 1 The Department of Urology, The Second Affiliated Hospital of Kunming Medical University, No. 374 Dian Mian Avenue, Kunming, 650101 Yunnan People’s Republic of China; 2 The Department of Oncology, The Second Affiliated Hospital of Kunming Medical University, Kunming, China; 3 The Department of Biochemistry and Molecular Biology, Kunming Medical University, Kunming, China

**Keywords:** Bladder carcinoma, Serine hydroxymethyltransferase, The differential expressed genes (DEGs), Extracellular matrix (ECM), Cell cycle, Bladder, Bladder cancer

## Abstract

Bladder cancer (BLCA) is a common malignant tumor in urinary system all over the world. However, due to its high recurrence rate and complex causes, clinicians often have limited options for surgical and drug treatments. Recent researchs on the molecular mechanism of BLCA have reveals its biological progress and potential for early diagnosis. Serine hydroxymethyltransferase 1/2 (SHMT1/2) is a crucial enzyme in the one-carbon metabolism of tumor cells, and the expression levels of these isozymes have been found to be associated with the biological progression of various malignant tumors. However, the impact of SHMT1/2 on the biological progression of bladder cancer and its molecular regulation mechanism remain unclear. In this research utilizes BLCA clinical sample data, the TCGA database, and in vitro cell experiments to predict the expression levels of SHMT1/2 in BLCA. The findings indicate that SHMT1 remained unchanged, while SHMT2 expression is increased in BLCA, which was related to poor prognosis. Additionally, SHMT2 affects the growth, migration, and apoptosis of bladder cancer cells in vitro. It also influences the expression levels of E-cadherin and N-cadherin, ultimately impacting the malignant biological progression of bladder tumors. These results establish a correlation between SHMT2 and the malignant biological progression of BLCA, providing a theoretical basis for the early diagnosis and treatment of bladder cancer.

## Introduction

Bladder cancer (BLCA) is a prevalent malignant tumor of the urinary system worldwide. It is predicted that in 2023, there will be 82,290 BLCA patients in the United States, resulting in an estimated death toll of 16,710^1^. The age-standardized incidence rate (ASIR) of BLCA is expected to continue rising in the next decade^2^. This complex and heterogeneous disease primarily originates from the epithelial cells of the inner wall of the bladder and is influenced by genetic and environmental factors^3^. Based on the classification of pathological features, approximately 70–75% of cases were diagnosed as non-muscle-invasive bladder cancer (NMIBC) and are treated with transurethral resection of bladder tumor (TURBT). The remaining 25–30% of patients have tumor cells that have invaded deeper layers of the bladder wall or formed metastases, known as muscle-invasive bladder cancer (MIBC). These patients have a poor prognosis, often with a mortality rate exceeding 50%^4^. The main treatment methods of MIBC patients include radical cystectomy (RC), chemotherapy, and radiotherapy^5^. However, the long-term survival rates of patients with bladder cancer has remained unchanged for decades.

Urinary biomarkers have been developed as potential alternatives or adjuncts to standard tests for the initial diagnosis of bladder cancer or identification of recurrent disease. The U.S. Food and Drug Administration (FDA) has approved six urinary biomarkers for the diagnosis or surveillance of bladder cancer: BTA stat (Polymedco), BTA TRAK (Polymedco), NMP22 (Matritech), NMP22 BladderCheck Test (Alere), Immuno-Cyt (Scimedx) and FISH (UroVysion). Some researchers reported that noncoding RNAs has potential to prognosis bladder cancer as biomarker, such as CircRNAs, LncRNAs and MiRNAs^6^. Additionally, a novel biomarker of bladder cancer has been found is exosome, numerous studies investigating the association between exosomes and bladder cancer have identified the presence of exosome MALAT1, PCAT-1, and PTENP1^7^.

Metabolic alterations in BLCA have become the focus of cancer research. The progress in malignant biology in BLCA is often accompanied by various metabolic reprogramming processes to maintain high proliferation. Serine, a crucial participant in the folic acid cycle, serves as an important single-carbon source for tumor cells. It also supports the biosynthesis of purine and pyrimidine, participates in the methionine cycle, methylation reactions, and the generation of NADPH for antioxidant defense and the growth and proliferation of cancer cells^8,9^. Although bladder cancer cells continuously acquire exogenous serine, they frequently undergo reprogramming of the serine/glycine conversion pathway. This reprogramming accelerates the breakdown of serine and produces more one-carbon units to support rapid growth^10,11^. The key enzyme in this pathway, Serine Hydroxymethyltransferase (SHMT), has been found to be upregulated in BLCA, promoting tumor cell proliferation and inhibiting apoptosis^12^.

However, the mechanisms by which SHMT regulates the growth of BLCA and its involvement in molecular processes remain unclear. Therefore, this study aims to investigate the differential expression levels of serine hydroxymethyltransferase in BLCA and normal bladder tissue and examine whether this difference can impact the malignant biological phenotype of bladder cancer in vitro. This research can provide a basis for further exploration of SHMT as a potential driver of BLCA, as well as confirm the correlation between SHMT2 and the malignant biological progression of BLCA.

## Materials and methods

### Pan-oncogene analysis: human malignancies express SHMT1 and SHMT2

TIMER 2.0 is a recently developed web server (http://timer.cistrome.org/) designed to analyze and estimate transcriptome data from tumor and normal samples sourced from the TCGA tumor database. It utilizes six different standard computational methods to provide efficient analysis of associations between gene expression, immune infiltration, mutations, and survival characteristics in the TCGA cohort ^13^.

### Bioinformatics analysis: SHMT1 and SHMT2 expression in bladder cancer

The Cancer Genome Atlas, Provisional^14^, is the largest gene expression database that contains sequencing and pathological data from 30 different cancers. To access the BLCA transcriptome data file, please visit their official website (https://portal.gdc.cancer.gov/). The file includes a tumor group (*n* = 412) and a normal group (*n* = 19). Using R language 4.1.3 and R toolkits 'limma' and 'ggplot2', we analyzed the BLCA transcriptome data files from the TCGA database. Specifically, we focused on distinguishing and analyzing the differences between the SHMT1 and SHMT2 genes in the tumor and normal groups.

### Survival analysis based on database

GEPIA is an interactive web server developed for the analysis of tumor and normal samples. It utilizes the RNA-Seq expression data from the Cancer Genome Atlas and Genotype Tissue Expression (GTEx) project, employing standard processing pipelines. GEPIA offers various customizable functions, including tumor/normal differential expression analysis, analysis based on cancer type or pathological stage, patient survival analysis, similar gene detection, and correlation analysis.

### Prediction of the SHMT2 downstream molecular pathway

The GO (Gene Ontology) database was used to define and describe the functions of genes and proteins from various species^15^. For GO enrichment analysis of differential genes, the R packages (clusterProfiler, org.Bt.eg.db, enrichplot) were utilized. The differential gene ID was mapped to the GO node information in the database, and a statistical map was created for the secondary classification of the expressed protein. Similarly, the KEGG (Kyoto Encyclopedia of Genes and Genomes) database, which systematically analyzes gene functions and links genomic information with functional information, was used^16^. The R packages (clusterProfiler, org.Bt.eg.db, enrichplot) were employed for KEGG pathway enrichment analysis of differential genes, with a filter condition of *P* < 0.05. The results were visualized and outputted as statistical graphs.

### Clinical sample

From July 2022 to January 2023, we obtained 10 groups of clinical specimens from different patients who diagnosed as MIBC and suitable for radical cystectomy from the Second Affiliated Hospital of Kunming Medical University. Each group consisted of bladder cancer tissue and their adjacent non-tumor tissue. The specimens were collected before radiotherapy or chemotherapy and stored in liquid nitrogen immediately after radical resection during surgery. All patients were diagnosed as MIBC by imaging and pathological examination before operation. All procedures were performed in accordance with the Declaration of Helsinki. All patients corresponding to the samples signed the informed consent form. And all experimental procedures related to patient samples have been reviewed and approved by the Ethics Committee of the Second Affiliated Hospital of Kunming Medical University (Approval NO. FEY-BG-39–2.0).

### Transcriptome sequencing and analysis for clinical specimens

#### RNA Isolation and library preparation

Of all clinical specimens, 5 groups were used for transcriptome sequencing. Total RNA was extracted using the TRIzol reagent (Invitrogen, CA, USA) according to the manufacturer’s protocol. RNA purity and quantification were evaluated using the NanoDrop 2000 spectrophotometer (Thermo Scientific, USA). RNA integrity was assessed using the Agilent 2100 Bioanalyzer (Agilent Technologies, Santa Clara, CA, USA). Then the libraries were constructed using VAHTS Universal V6 RNA-seq Library Prep Kit according to the manufacturer’s instructions. The transcriptome sequencing and analysis were conducted by OE Biotech Co., Ltd. (Shanghai, China).

#### RNA sequencing and differentially expressed genes analysis

The libraries were sequenced on an llumina Novaseq 6000 platform and 150 bp paired-end reads were generated. Raw reads of fastq format were firstly processed using fastp^17^ and the low quality reads were removed to obtain the clean reads. The clean reads were mapped to the reference genome using HISAT2^18^. FPKM^19^ of each gene was calculated and the read counts of each gene were obtained by HTSeq-count^20^. PCA analysis were performed using R (v 3.2.0) to evaluate the biological duplication of samples.

Differential expression analysis was performed using the DESeq2^21^. Q value < 0.05 and foldchange > 2 or foldchange < 0.5 was set as the threshold for significantly differential expression gene (DEGs). Hierarchical cluster analysis of DEGs was performed using R (v 3.2.0) to demonstrate the expression pattern of genes in different groups and samples. The radar map of top 30 genes was drew to show the expression of up-regulated or down-regulated DEGs using R packet ggradar.

GO and KEGG pathway analysis methods are the same as those used in bioinformatics analysis. The mRNA sequencing raw data generated and analyzed during the current study is available in the NCBI SRA repository (Sequence Read Archive; https://www.ncbi.nlm.nih.gov/sra/PRJNA999182). The corresponding author can be contacted for more information on these mRNA sequencing data presented herein.

#### Culture of cells

A cell culture incubator set at 37 °C with 5% CO2 was used to cultivate human bladder metastatic cell carcinoma cells J82 from the Kunming Institute of Animal Science, Chinese Academy of Sciences, in DMEM/F12 medium with 10% FBS (Thermo Fisher) and 1% double antibodies. McCoy's 5A medium (Thermo Fisher) was used to cultivate human bladder metastatic cell carcinoma cells T24 (Beijing Beina Chuanglian Institute of Biotechnology), with the remainder of the culture conditions remaining the same as for J82.

#### Cellular transfection

T24 and J82 cells were seeded in 6-well plates at a density of 2 × 10^5^ cells per well and cultured overnight in an incubator. The 6-well plate was labeled with grouping information. The 'empty group' consisted of 250 μL serum-free medium opti-MEM + 3.5 μL Lipofectamine 3000 + 7 μL NC siRNA. The 'transfection group' consisted of 250 μL serum-free medium opti-MEM + 3.5 μL Lipofectamine 3000 + 7 μL SHMT2 siRNA. The transfection solution was prepared according to the detailed grouping information, vortexed and shaken for 15 min, and then 250 μL of the transfection solution was added to each well. The medium did not need to be changed every other day, and after 48 h, the cell density would reach 80%-90% for further processing. The si-RNA sequence used was as follows: Si-SHMT2: forward, 5′′-GGAGAGUUG UGGACUUUAUTT-3′; reverse, 3′-TTCCUCUCA ACACCUGAAAUA-5′.

#### Cell scratching

The following steps were performed in the experiment: (1) Marking: Using a marker pen with a ruler, evenly draw at least 5 lines in each well of the 6-well plate. The marking lines should be evenly spaced. (2) Spreading: Inoculate 0.5 × 105 cells per well on the 6-well plate. (3) Scratch: After 24 h, observe the state of the cells. Use a 200 μl pipette tip to scratch perpendicular to the horizontal line on the back. Apply vertical force evenly, wash the cells twice with PBS, and add 2 ml of serum-free medium for further culturing. (4) Taking photos: Capture photos of the samples at 0 and 24 h. (5) Result analysis: Calculate the cell migration rate (wound healing rate) using the formula: (initial scratch area—scratch area at time t) / initial scratch area.

### Cell cycle measured by flow cytometry

The transfected J82 and T24 cells were digested and transferred to a centrifuge tube. They were then centrifuged at 1500 rpm for 5 min. After centrifugation, the cells were resuspended with 300 ul of PBS (containing 2% FBS) and added dropwise to 5 ml of pre-cooled 70% ethanol. The cells were fixed overnight at 4 °C. The next day, the cell samples were collected by centrifugation, washed twice with PBS, and centrifuged again. 500 ul of PI/RNase Staining Buffer was added and the samples were protected from light for 30 min. Cell cycle changes were detected using a FACSCelesta 3 laser flow cytometer with an excitation wavelength of 488 nm and an emission wavelength of 620 nm. The experimental data were recorded.

### Apoptosis measured by flow cytometry

The transfected cells were trypsinized, centrifuged, and then washed in PBS. Next, 500 ul of 1X Buffer was added to resuspend the cells. Each sample was then treated with 5 ul of Annexin V-633 and 5 ul of PI, and incubated in the dark for 30 min. Flow cytometry analysis was performed using a flow cytometer with an excitation light wavelength of 488 nm. FITC fluorescence was detected using a passband filter with a wavelength of 515 nm, while PI was detected using another filter with a wavelength greater than 560 nm. The experimental results were recorded, and the proportion of apoptotic cells was determined. The calculation formula used was: percentage of apoptotic cells = percentage of early apoptotic cells (positive for Annexin V-633 staining and negative for PI staining) + percentage of late apoptotic cells (positive for Annexin V-633 staining and positive for PI staining).

### Western blot

The transfected J82 and T24 cells were lysed using protein inhibitor modified RIPA lysate (Thermo Fisher) on ice, and the total cell protein was extracted. After accurate loading, electrophoresis separation was performed on a 10% SDS-PAGE gel, and then transferred to a superior PVDF membrane. The PVDF membrane was blocked with skim milk (BD) for 2 h. The following primary antibodies were added and incubated overnight at 4 °C, anti-SHMT2(1:1,000, ab224428; Abcam, Cambridge, UK), anti-Tubulin(1:10,000, ab176560; Abcam, Cambridge, UK), anti-MMP7(1:1,000, ab205525; Abcam, Cambridge, UK), anti-Integrin β-4(1:1,000, ab182120; Abcam, Cambridge, UK), anti-Laminin β-4(1:500, Sc-130540; Santa Cruz, CA, USA), anti-SHMT1(1:800, Cat.14149–1-AP; Proteintech, Wuhan, China), anti-E-cadherin (1:2,000, Cat.60335–1-Ig; Proteintech, Wuhan, China), anti-N-cadherin (1:2,000, Cat.66219–1-Ig; Proteintech, Wuhan, China), anti-MMP2 (1:2,000, Cat.66366–1-Ig; Proteintech, Wuhan, China), anti-GAPDH (1:10,000, Cat.60004–1-Ig; Proteintech, Wuhan, China). The membrane was washed the next day and secondary antibodies were added and incubated for 30 min. After washing, the membrane was treated with ECL chemiluminescence reagent for 1 min and finally imaged using a fluorescence imaging system. The gray value of protein bands was analyzed using Image J software and the experimental data were recorded.

### Immunohistochemistry

Clinically collected human bladder cancer tissues and paracancerous tissues were dehydrated and fixed. They were then dewaxed in xylene for 20 min twice, rehydrated with graded alcohol (100% × 2, 95%, 90%, 85%, distilled water, 5 min each time), followed by antigen retrieval using the heating method. To eliminate endogenous peroxidase, 3% hydrogen peroxide was used. The tissues were blocked with the same species of secondary antibody serum, incubated overnight at 4 °C with the primary antibody, and then incubated with the secondary antibody. DAB color development was performed, followed by hematoxylin counterstaining, dehydration and transparency, and neutral gum sealing.

### Statistical analysis

The experimental results were expressed as Mean ± SEM. Statistical analysis of the experimental data was performed using GraphPad Prism version 8. For single-group experimental data, a two-sided unpaired *t*-test was conducted to analyze the differences. For multiple experimental data, one-way ANOVA was used for statistical analysis. A significance level of *P < 0.05 was considered to indicate a significant difference.

### Ethical statement

This study and all human bladder cancer specimens in the study were collected and experimented on in strict accordance with the requirements of the committee. Approval number FEY-BG-39–2.0.

## Results

### Expression of SHMT1 and SHMT2 in 23 kinds of tumor and bladder cancer

**Figure 1:**
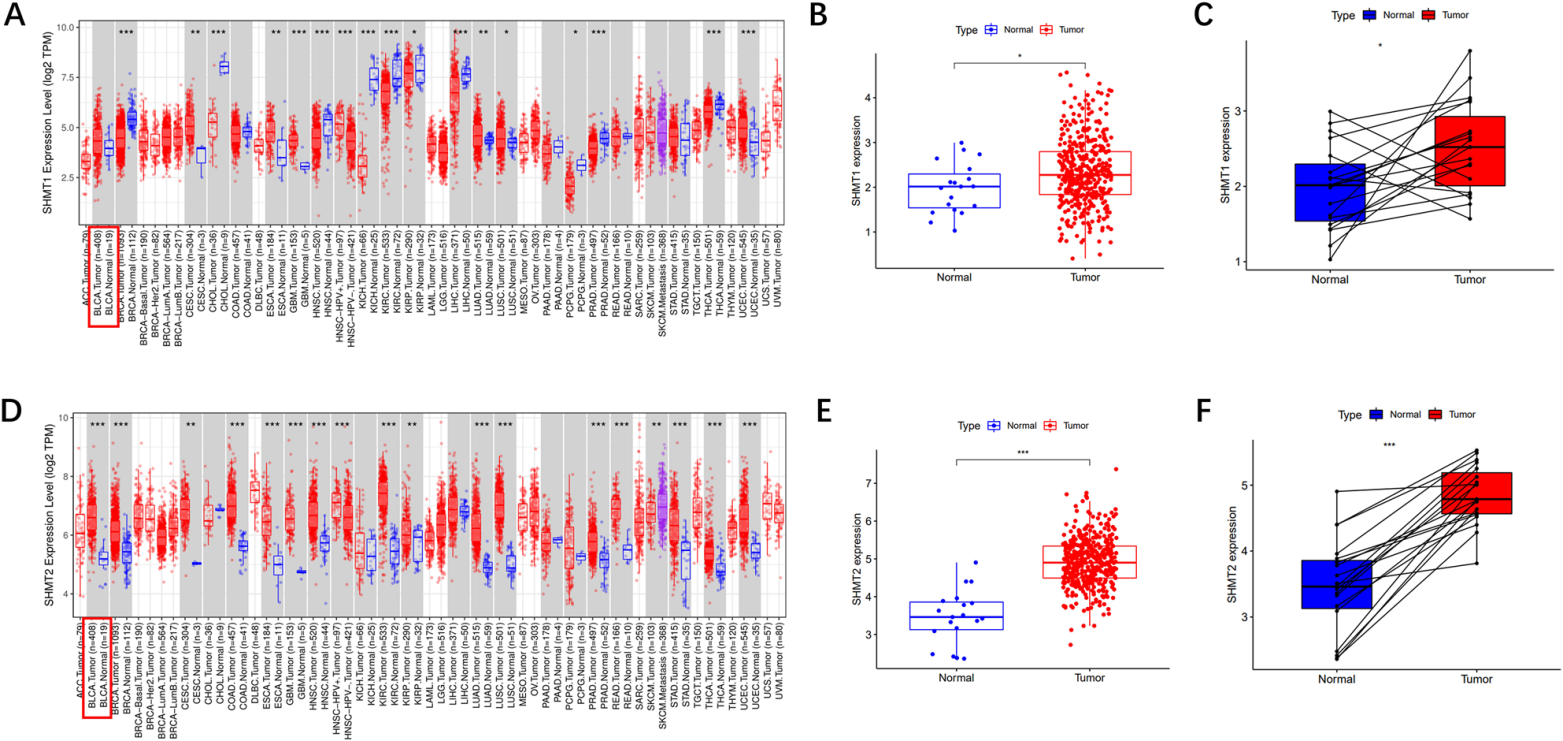
The expression levels of SHMT1 and SHMT2 in 23 tumor tissues and bladder cancer. (**A**,**D)**: The expression levels of SHMT1 and SHMT2 in 23 tumor tissues are shown using the TIMER 2.0 server (http://timer.cistrome.org/), with BLCA data expression levels highlighted in red; (**B** and **E)** are data samples from the same source that combine SHMT1/2 expression; (**C** and **F)** are data from the tumour group with those from the normal group derived from the same patient to obtain paired difference analysis (*n* = 19 in the tumour group, *n* = 19 in the normal group), * *P* < 0.05, ** P < 0.01, *** P < 0.001.

TIMER 2.0 output gene pan-cancer difference analysis between the two groups is shown in Fig. 1A. There is no significant difference in the expression of SHMT1 in BLCA in the red box (*P* > 0.05). Using R-4.1.3 software, the BLCA transcriptome data in TCGA was analyzed, revealing that the expression of SHMT1 in bladder cancer was significantly higher than that in normal tissues (*P* < 0.05), as shown in Fig. 1B. Further analysis using the 'paired difference analysis' method showed that the expression of SHMT1 in the tumor group was also significantly higher than that in the normal group (*P* < 0.05), as depicted in Fig. 1C. The analysis results of the three groups for SHMT1 showed differences, which will be discussed later. In the subsequent analysis of the SHMT2 gene using the same method (Fig. 1D, E, F), it was found that the expression of SHMT2 in bladder cancer was significantly higher than that in normal tissue, and the results of the three groups exhibited the same trend (*P* < 0.001).

### Significance of SHMT1/2 expression difference on survival analysis and pathological grading of bladder cancer in database

**Figure 2:**
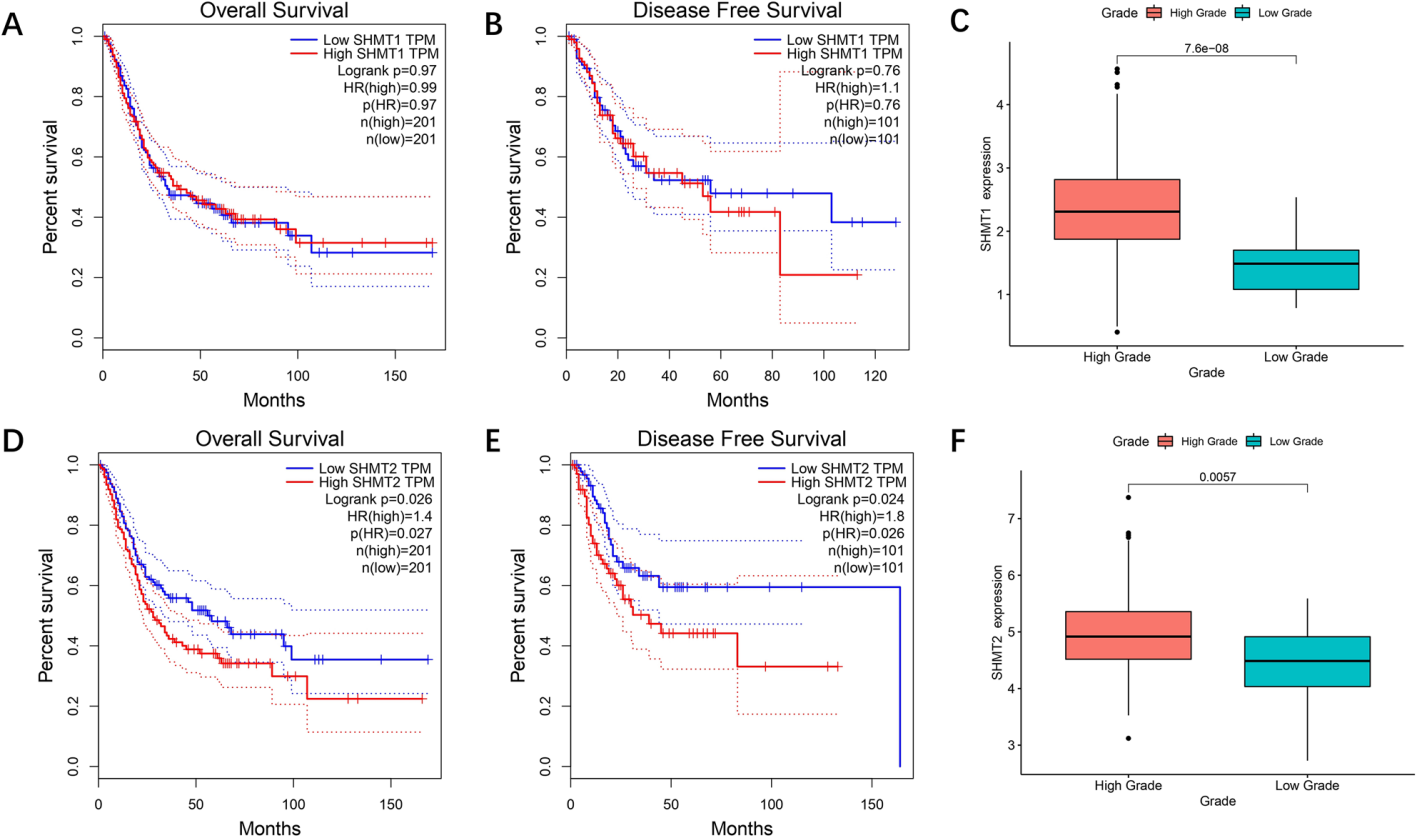
Analysis of the correlation between the clinical grading for SHMT1 and SHMT2 and clinical survival time. (**A**, **D**) GEPIA server-generated SHMT1 and SHMT2 OS curves; (**B**,**E**): GEPIA server-visualized SHMT1 and SHMT2 DFS curves; (**C**,**F**): clinical grade of each BLCA sample by R language Grade data were combined with their SHMT1/2 expression to visualise the results of clinical grade and gene correlation analysis, * *P* < 0.05, ** P < 0.01, *** P < 0.001.

The GEPIA server was used to generate overall survival (OS) and disease free survival (DFS) for SHMT1 and SHMT2. The OS analysis showed a decrease in the survival rate over time for both the high and low expression groups of SHMT1, but there was no significant difference between the two groups (Fig. 2A  *P* > 0.05). Similarly, the DFS analysis showed a significant decrease in the survival rate over time for both groups, but no significant difference between them (Fig. 2B, P > 0.05). The correlation between clinical grade and gene expression was analyzed using the R language toolkit. The expression level of SHMT1 was found to be higher in the high grade group compared to the low grade group (Fig. 2C, *** *P* < 0.001).The same analysis was performed for the SHMT2 gene. Both OS and DFS analyses showed a decrease in the survival rate over time for both the high and low expression groups of SHMT2, with the low expression group having a better overall survival rate compared to the high expression group. The difference between the two groups was significant (Fig. 2D and Fig. 2E, * *P* < 0.05, * *P* < 0.05). In the clinical grade correlation analysis, the expression level of SHMT2 was significantly higher in the high-grade group compared to the low-grade group (Fig. 2F, *** *P* < 0.001).

### Gene enrichment analysis based on transcriptome sequencing results of clinical MIBC specimens and TCGA dataset

**Figure 3:**
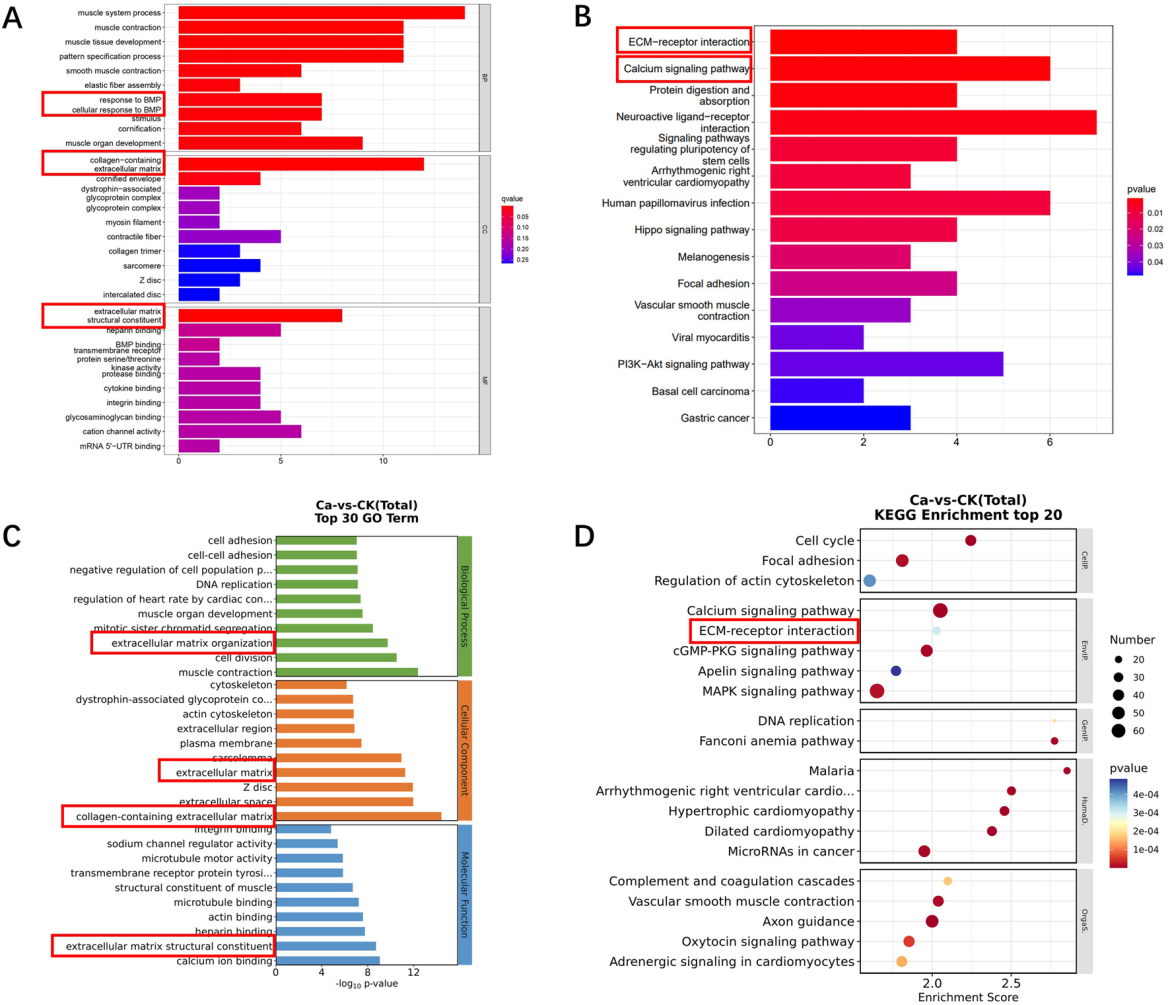
Analysis of bladder cancer using GO enrichment and KEGG enrichment from various data sources. (**A**): The number of GO entries corresponding to the enrichment is represented by the number of entries on the horizontal axis at the bottom of the graph; the higher the number of entries means the stronger the correlation, and the smaller the *P*-value, the redder the color, the more significant the difference. * *P* < 0.05; The number of GO entries corresponding to the (**C**): transcriptome sequencing GO enrichment analysis, the smaller the *P* value, the redder the color, the more significant the difference; the larger the circle, the more enriched entries. (**B)**: KEGG enrichment analysis generated by R language toolkit. The smaller the *P*-value, the redder the color, the more significant the difference. Pairs of combined correlations are represented by numbers on the horizontal axis at the bottom of the graph; the higher the score, the more dependable the data.

According to R-4.1.3, we conducted an analysis of the BLCA transcriptome samples from the TCGA database. This analysis focused on GO enrichment analysis, specifically in the areas of biological process (BP), cellular component (CC), and molecular function (MF) to elucidate the role of the SHMT2 gene. Figure 3A illustrates the findings of the GO enrichment analysis for the biological process category, indicating potential associations between SHMT2 and serine/threonine kinase receptor (BMP receptor, BMPR), muscle contraction, and muscle tissue development. In terms of molecular function, SHMT2 is predicted to be involved in extracellular matrix and cytoplasmic membrane keratinization. Additionally, the analysis suggests that SHMT2 has a role in the structural components of the extracellular matrix (*P* < 0.05). KEGG enrichment analysis revealed that the main enriched signaling pathways associated with SHMT2 are the extracellular matrix receptor (ECM-receptor), calcium ion signaling pathway, and stem cell signaling pathway, as shown in Fig. 3B (*P* < 0.05).

As described in materials and methods, we collected 10 groups of MIBC specimens during radical cystectomy. The pathological results of 10 cases included in the study after radical cystectomy were all high-grade urothelial carcinoma. Transcriptome sequencing was performed on 5 groups of specimens. The basic results of transcriptome sequencing are shown in the supplementary material 1, and the original data of sequencing can also be found in NCBI SRA repository (PRJNA999182). In the GO enrichment analysis (Fig. 3C), it was observed that MIBC is associated with cell adhesion, DNA replication, and muscle contraction in terms of biological processes. Regarding cell localization, the analysis suggests a potential relationship with extracellular matrix proteins, cytoskeleton, and the extracellular environment. In terms of molecular function (Fig. 3C), MIBC is predicted to be related to integrin, calcium ion, and heparin. The KEGG enrichment analysis (Fig. 3D) highlighted the calcium signaling pathway and the extracellular matrix receptor (ECM-receptor) as the main enriched signaling pathways. These findings suggest that the calcium signaling pathway, ECM-receptor, and stromal receptor-related pathways play a crucial role in the molecular pathways affecting the progression of MIBC.

### Expression of SHMT1 and SHMT2 in clinical specimens

**Figure 4:**
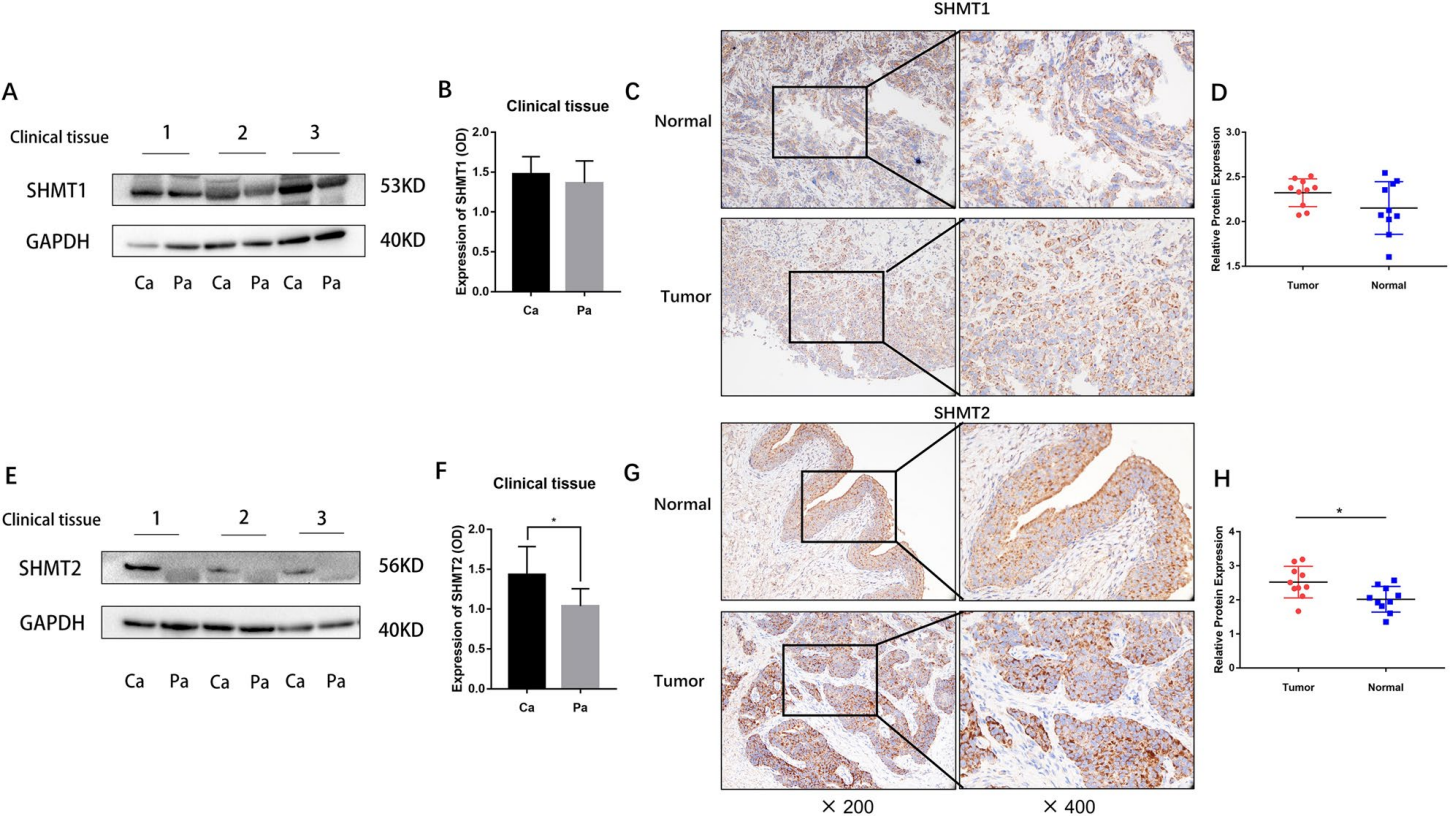
Western blotting to detect the expression of SHMT1 and SHMT2 in clinical samples. (**A** and **E)**: Western blot to determine the levels of SHMT1 and SHMT2 expression in clinical BLCA tissue specimens; (**B** and **F)**: Statistical analysis of the data using ImageJ for the band’s grey scale values and GraphPad Prism version 8 for the histogram, with unpaired *t*-test (*n* = 3), * *P* < 0.05, ** P < 0.01, *** P < 0.001. (**C** and **G)**: Immunohistochemical detection of SHMT1 and SHMT2 expression levels in clinical BLCA tissue specimens; (**D** and **H**): ImageJ analysis of photographs to determine the target gene expression; GraphPad Prism version 8 for the histogram; statistical analysis using unpaired *t*-test (*n* = 3), * *P* < 0.05, ** P < 0.01, *** P < 0.001.

The expression levels of SHMT1 and SHMT2 proteins in our clinical MIBC tissues were detected using Western Blot and immunohistochemical. The results showed that there was no significant difference in the expression of SHMT1 in clinical tissues (*P* > 0.05) (Fig. 4A, B, C and D). However, the expression of SHMT2 in clinical tissues of bladder cancer was found to be higher than that in corresponding paracancerous tissues (*P < 0.05) (Fig. 4E, F, G and H). Considering the existing data, previous bioinformatics analysis, and transcriptome sequencing results, it can be inferred that SHMT2, one of the two isozyme genes of serine hydroxymethyltransferase, is more likely to be a potential driver of bladder cancer than SHMT1.

### ECM-related protein expressions in clinical specimens

**Figure 5:**
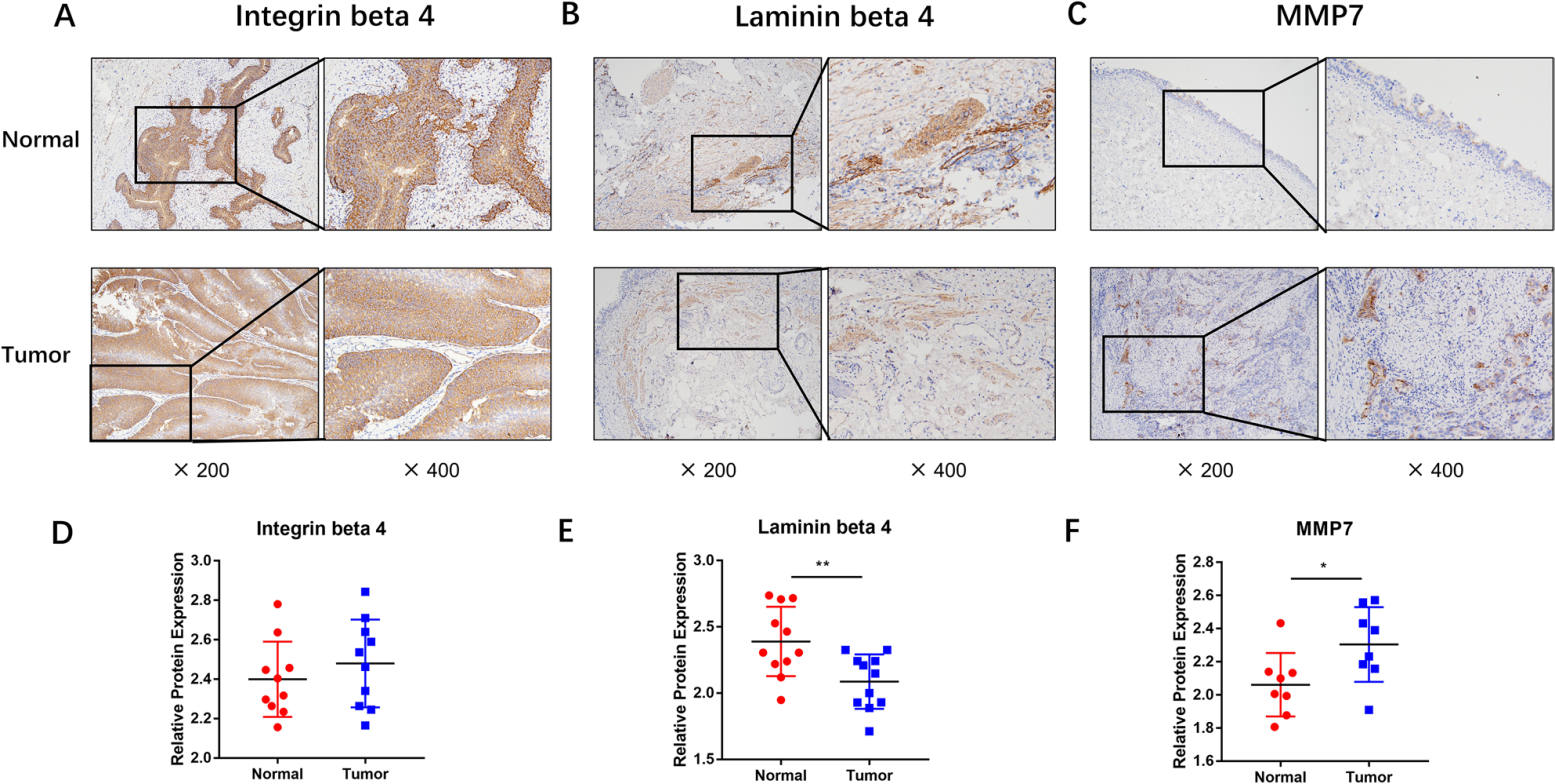
Immunohistochemical detection of expression of ECM-related genes in clinical samples. (**A**,**B**,**C**): Immunohistochemical detection of expression of Integrin beta 4, Laminin beta 4 and MMP7 in clinical BLCA tissue specimens; (**D**,**E**,**F)**: ImageJ analysis of photographs to obtain the expression of corresponding target genes, GraphPad Prism version 8 for histogram, unpaired *t*-test was used to statistically analyse the results (*n* = 3), * *P* < 0.05, ** P < 0.01, *** P < 0.001.

The expression of ECM-related molecule Integrin beta 4, Laminin beta 4 and MMP7 in our clinical MIBC tissues was examined through immunohistochemical experiments. The results presented in Fig. 5A and D indicated that there was no significant difference in its expression (*P > 0.05), which aligned with the findings from transcriptome analysis. However, the expression level of Laminin beta 4 in MIBC was found to be down-regulated in tissues compared to adjacent tissues (**P < 0.01) (Fig. 5B and E). On the other hand, Fig. 5C and F demonstrated an increased expression level of MMP7 in MIBC compared to adjacent tissues (*P < 0.05).

### Inhibition of SHMT2 gene expression changes the malignant biological phenotype of urothelial cancer cells in vitro

**Figure 6:**
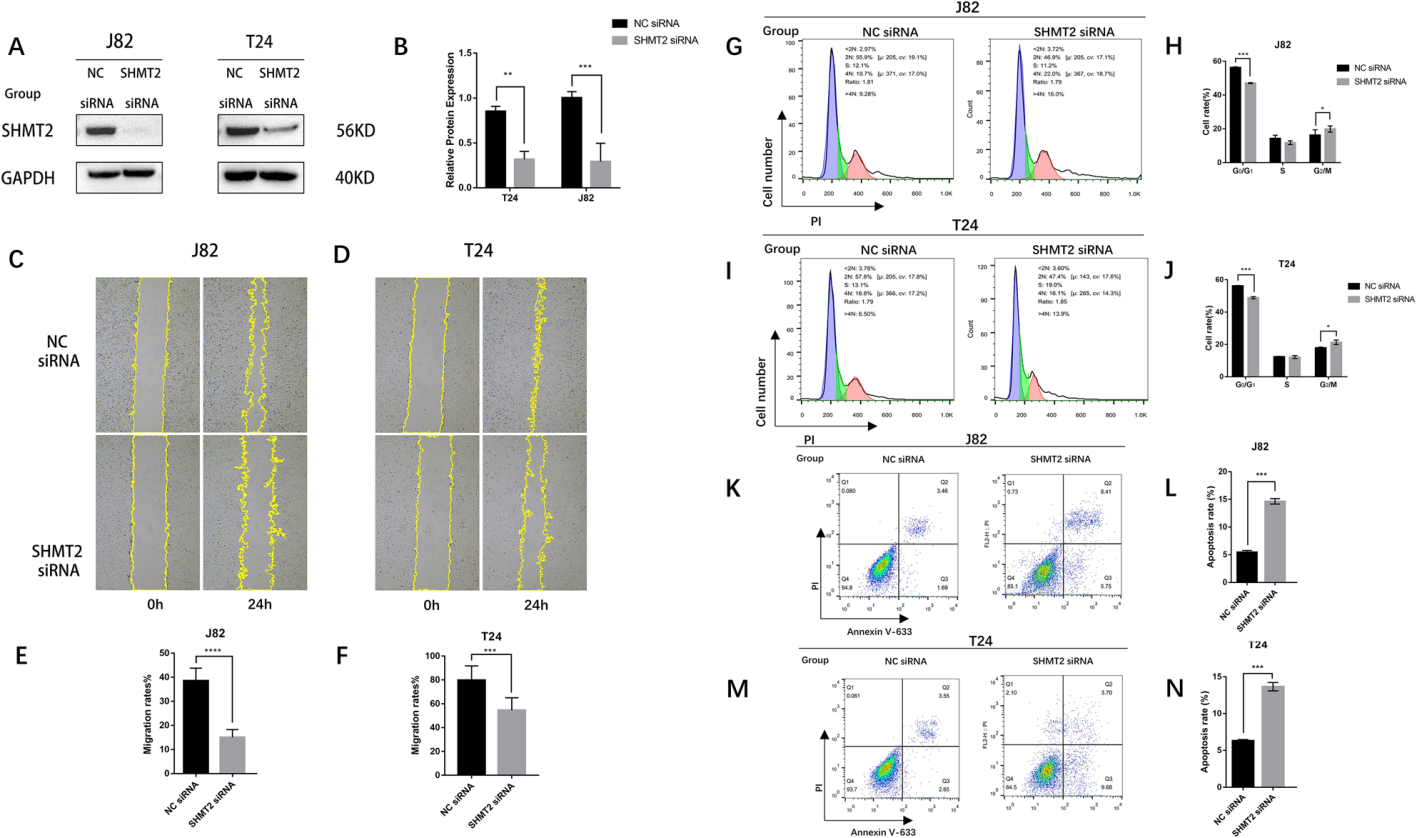
After siRNA interference with SHMT2 gene expression, bladder cancer cells exhibit altered malignant biological capabilities. (**A)** Western Blot to measure the level of SHMT2 expression in J82 and T24 cells; (**C** and **D)**: transfected treated T24 and J82 cells were scratched, photographed, and ImageJ was used to assess the migration rate; (**G**,**H)**: PI staining, flow cytometry, and SHMT2 siRNA transfected treated J82 and T24 for 48 h; (**K**,**M)**: After transfecting SHMT2 siRNA, apoptotic alterations were detected using flow cytometry; Q1: Cells with mechanical injury or nuclei that are bare or stained negatively for annexin V-633 or stained positively for PI; Q2: Late apoptotic cells that show positive PI and Annexin V-633 staining; Q3: PI staining is negative while AnnexinV-633 staining is positive (**H**,**J**,**L**,and **N)**: statistical data, followed by a histogram created with GraphPad Prism version 8 and statistical analysis using a two-sided unpaired *t*-test (*n* = 3), with *P* < 0.05, 0.01, 0.001 for significance.

Based on previous experimental results, the SHMT2 gene expression level was reduced using the transient transfection method in vitro. The knockdown effect is illustrated in Fig. 6A and B. Compared to the control group, the expression levels of SHMT2 gene in J82 and T24 cells were decreased by approximately 70% and 60% (***P < 0.001, **P < 0.01), respectively. Subsequent cell scratch experiments, as shown in Fig. 6C, D, E, F, revealed that the 24-h migration rate of J82 cells in the control group was 38.62%, which was significantly higher than the migration rate of the transfection group (15.04%) (*** *P* < 0.001). Similarly, the 24-h migration rate of T24 cells in the control group (79.76%) was greater than that in the transfection group (54.53%) (***P < 0.001). These findings suggest that the decrease in SHMT2 expression level can impede the migration ability of bladder cancer cells J82 and T24.

The cell cycle was analyzed using flow cytometry. In Fig. 6G and H, it can be observed that compared to the empty group, the proportion of cells in the G0/G1 phase decreased to 47.1% (*** *P* < 0.001) in J82 cells. Additionally, the proportion of cells in the S phase decreased to 11.8%, while the proportion of cells in the G2/M phase increased to 19.87% (* *P* < 0.05). Similarly, in Fig. 6I and J, the proportion of cells in the G0/G1 phase decreased to 48.9% (*** *P* < 0.001) in T24 cells. The proportion of cells in the S phase decreased to 12.2%, and the proportion of cells in the G2/M phase increased to 21.3% (* *P* < 0.05). These results indicate that reducing the expression level of SHMT2 leads to a decrease in the proportion of cells in the G0/G1 phase and an arrest of the cell cycle in the G2/M phase in bladder cancer cells J82 and T24. Cell apoptosis was also examined using flow cytometry. In Fig. 6K and L, it was observed that the 24-h migration rate of J82 cells in the empty group was 38.62%, which was significantly higher than the migration rate in the transfection group (15.04%) (*** *P* < 0.001). Similarly, in Fig. 6M and N, the 24-h migration rate of T24 cells in the empty group was 79.76%, while the migration rate in the transfection group was 54.53% (*** *P* < 0.001). These findings suggest that reducing the expression level of SHMT2 inhibits the migration ability of bladder cancer cells J82 and T24.

### Inhibition of SHMT2 gene expression changes the expression of proteins related to migration function of urothelial cancer cells in vitro

**Figure 7:**
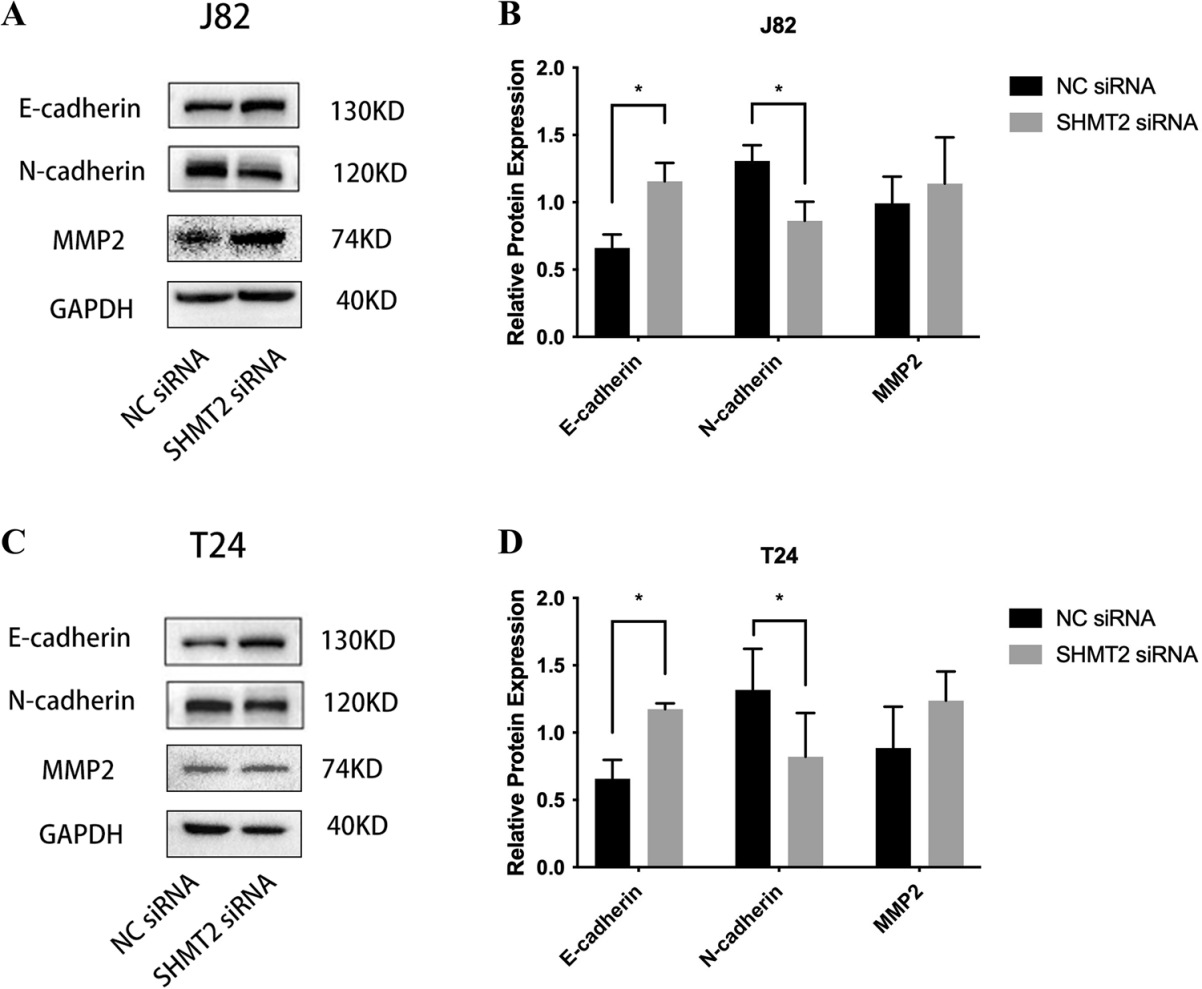
How the expression of the invasion-related gene SHMT2 was altered in human J82 and T24 bladder cancer cells after siRNA interference. (**A**, **B**): Western blot analysis after transfection revealed altered expression of E-cadherin, N-cadherin, and MMP2; (**C**, **D**): GraphPad Prism version 8 was used to create a histogram; the results were statistically analyzed using an unpaired *t*-test (*n* = 3), * *P* < 0.05, ** P < 0.01, *** P < 0.001.

After transient knockdown of the SHMT2 gene expression, Western Blot was performed to detect changes in invasion-related genes E-cadherin, N-cadherin, and MMP2 in bladder cancer cells T24 and J82. The results are presented in Figs. 7A and B. In J82 cells, the expression level of the SHMT2 gene decreased by approximately 60% compared to the control group, which was statistically significant (*P < 0.05). Similar results were observed in T24 cells as well. The expression level of the SHMT2 gene decreased by approximately 70% in T24 cells compared to the control group, which was statistically significant (*P < 0.05) (Fig. 7C and D).

## Discussion

Serine Hydroxymethyltransferase (SHMT) is a key protein involved in the decomposition and synthesis of serine/glycine and one-carbon metabolism. In humans, SHMT consists of two isozymes: the cytoplasmic isozyme (SHMT1) and the mitochondrial isozyme (SHMT2). SHMT1 primarily resides in the cytoplasm and catalyzes the breakdown of serine to provide a one-carbon unit for nucleotide synthesis, influencing gene methylation and DNA synthesis^22^. Previous studies have demonstrated that the reduction of SHMT1level can hinder the growth of tumor ^23^. SHMT1 affects the expression of downstream molecules ROS through NOX1 in hepatocellular carcinoma, leading to the production of EMT and MMP2 and ultimately influencing its proliferation^24^. In lung adenocarcinoma, it reverses the killing effect of NK cells on cancer cells, and promotes the progress of cancer ^25^. SHMT2, primarily located in mitochondria, is also a crucial enzyme in one-carbon unit metabolism^26^. In prostate cancer cells, the expression of SHMT2 is upregulated under the regulation of the typical JAK2/STAT3 pathway, affecting the early inflammatory response of cancer^27^. SHMT2 can participate in the interaction between transcription factor TCF4 and β-catenin, enhancing its own expression and promoting the growth and metastasis of colorectal cancer cells^28^.

In order to investigate the potential role of SHMT1 and SHMT2 as cancer drivers in BLCA, we conducted a data analysis using BLCA samples from the TCGA database^29^. In the pan-cancer analysis, there was no significant difference in the expression level of SHMT1. However, in the single-gene differential analysis, a significant difference was observed, which could be attributed to the variation in the number of BLCA samples between the two groups (*n* = 408 in pan-cancer analysis and *n* = 412 in single-gene differential analysis). Nevertheless, when considering the results of SHMT1 immunohistochemistry in clinical samples, the expression level of SHMT1 in BLCA was found to be not significantly different from that in normal bladder tissue. This indicates that SHMT1 cannot be considered as a potential cancer driver in BLCA. On the other hand, the expression level of SHMT2 in BLCA was significantly higher compared to normal bladder tissue (*P < 0.05, ***P < 0.001), and BLCA patients with high SHMT2 expression had significantly lower clinical survival time. Similar findings have been reported in studies on head and neck cancer and colorectal cancer samples from the TCGA database^30^. Therefore, among the two serine hydroxymethyltransferase isozymes, only SHMT2 shows a significant difference in expression between BLCA and normal tissues, suggesting its involvement in the biological progression of BLCA.

To investigate the metabolic mechanism of SHMT2 in BLCA, we conducted GO enrichment analysis using transcriptome sequencing of BLCA clinical samples and two sets of data from the TCGA database (Fig. 3). The results indicated that SHMT2 is associated with the structural components of the extracellular matrix. In the KEGG enrichment analysis, we found that there is a strong correlation between SHMT2 and the ECM-receptor interaction pathway, which indicates the potential connection. However, it is necessary to design additional experiments for further verification. We performed immunohistochemical experiments on clinical BLCA samples to examine the expression of Integrin beta 4, Laminin beta 4, and MMP7. We observed that there was no significant change in the expression of Integrin beta 4, which plays a key role in cell-ECM communication. However, the expression levels of laminin beta 4 and matrix metalloproteinase MMP7 were significantly altered. As our understanding of SHMT2 in BLCA is still limited, further discussion is required to determine if SHMT2 directly mediates ECM receptor-related molecules and regulates the malignant progress of BLCA.

In this study, we established an in vitro experimental model by transient interfering with the expression of SHMT2 in human bladder transitional cell carcinoma cells J82 and T24 using siRNA. Through the scratch test, we observed that the migration ability of BLCA cells weakened as the SHMT2 expression level decreased (the migration rate of J82 cells in the transfection group was 2.5 times lower than that of the non-transfection group, and the migration rate of T24 cells was 1.5 times lower, *** *P* < 0.001). Another study on SHMT2 also demonstrated that reducing the expression level of SHMT2 can inhibits the migration and growth of BLCA cells in vitro, which is consistent with our experimental results^12^. Therefore, maintaining a high expression level of SHMT2 is very important for the migration of BLCA cells in vitro. In our further study, we conducted flow cytometry analysis to investigate the changes of the cell cycle after SHMT2 expression of siRNA knockdown. Our findings revealed that a decrease in SHMT2 expression level leads to the decrease in the proportion of cells in the G0/G1 stage of bladder cancer cells. In addition, it was observed that the cell cycle was blocked in the G2/M phase, which affected the proliferation of BLCA cells. Another study also reported that silencing of SHMT2 led to a reduction in the protein expression of cell cycle regulators cyclinD1 and CDK4, causing cells to arrest in the G1 phase of the cell cycle^31^. These findings highlight the role of SHMT2 in influencing the BLCA cell cycle and controlling cell proliferation. Flow cytometry was used to observe the effect of siRNA knockdown of SHMT2 expression on apoptosis. The results demonstrated that a decrease in SHMT2 gene expression level led to an increase of apoptosis rate of BLCA cells. In clear cell renal cell carcinoma, siRNA knockdown of SHMT2 expression disrupted mitochondrial structure and function, reduced one-carbon metabolism, overactivated the autophagy-lysosomal pathway, resulting in an increase in LMP, and ultimately led to apoptosis^32^. Similarly, in another study on BLCA, loss of SHMT2 triggered ROS-dependent mitochondria-mediated apoptosis^33^. In conclusion, the SHMT2 gene plays a role in the malignant biological progress of bladder cancer. High expression levels of SHMT2 promote the proliferation and migration of malignant bladder tumor cells in vitro while inhibiting tumor cell apoptosis.

As a member of the matrix metalloproteinase (MMPs) family, MMP2 plays a significant role in degrading various components of the extracellular matrix. It facilitates the penetration of cancer cells into the basement membrane and is crucial in regulating tumor invasion and metastasis^34^. In an in vitro experimental model of SHMT2 transient knockdown, Western Blot analysis showed that the change in MMP2 expression was not statistically significant when compared to the control group. E-cadherin and N-cadherin, belonging to the cadherin family, are the most well-known members. These cadherins possess diverse structures and functions, and their interactions can have complex and diverse effects on biological processes^35^. In Fig. 7, it was observed that as SHMT2 decreased, the expression of E-cadherin increased and the expression of N-cadherin decreased. This means that the increase in E-cadherin and decrease in N-cadherin inhibited the invasion ability of bladder tumor cells T24 and J82. Another study on BLCA found that knocking down the H19 gene increased the expression of E-cadherin, affecting tumor cell proliferation and epithelial-mesenchymal transition, ultimately leading to the inhibition of BLCA's invasive ability ^36^. Additionally, for BLCA patients, higher N-cadherin expression was associated with a poorer recurrence-free survival rate in 40% of cases, and increased N-cadherin levels were often accompanied by greater tumor cell invasion^37^. Therefore, E-cadherin and N-cadherin may play a role in the regulatory network of SHMT2. As SHMT2 expression is up-regulated, E-cadherin expression increases and N-cadherin expression decreases, thereby impairing the invasion ability of bladder cancer cells.

The decrease in SHMT2 expression level has been found to weaken the in vitro migration ability and decrease the proliferation ability of bladder malignant tumor cells. Additionally, it has been observed to increase cell apoptosis, ultimately impacting the biological progress of bladder tumors. Notably, E-cadherin and N-cadherin may play a role in the SHMT2 regulatory network and influence the invasion ability of bladder cancer cells as SHMT2 expression changes. Regrettably, the research potential of this gene has not been fully realized in our current research progress, especially regarding the mechanism of interaction between SHMT2 and ECM receptors in the progression of bladder cancer. This aspect also constitutes one of our primary research objectives for the future. We aim to explore the mechanism of the gene and the ECM regulatory network by continuously investigating downstream interaction molecules of SHMT2. By uncovering the biological advancements of malignant bladder tumors, this research also offers novel insights for early diagnosis.

The application of non-coding RNAs in the diagnosis and treatment of bladder cancer has remained a contentious yet valuable area of research. Jiang et al. reported a panel of six miRNAs for diagnosing BLCA, with the six-miRNA panel achieving an AUC of 0.899 (95% CI, 80.00% sensitivity, 89.09% specificity)^6^. The upregulation or downregulation of biomarkers is a crucial factor in diagnosis. Therefore, the upregulated SHMT2 exhibits potential as a diagnostic biomarker for BLCA.

There are limitations to our present study. One major limitation is the short follow-up period, which prevented us from obtaining reliable survival data. Moreover, all cases were muscle-invasive bladder cancers, limiting the generalizability of our findings to non-muscle-invasive disease. While this homogeneity allowed for stable SHMT1/2 expression analysis, the expression pattern in non-muscle-invasive cases remains unvalidated. Longer follow-up and inclusion of non-muscle-invasive cases are needed.

## Conclusion

Upon combining all the information from this study, it becomes evident that SHMT2 plays a significant role in the onset of BLCA. The expression level of SHMT2 increased, and which was found to be negatively associated with patient survivability. In vitro experiments demonstrated that bladder malignancy cells exhibited reduced motility when SHMT2 expression levels decreased. In addition, it was observed that the low levels of SHMT2 expression blocked the bladder tumor cell cycle in the G2/M phase, which lead to decreased of cell proliferation and increased apoptosis. This resulted in a decrease in E-cadherin expression and an increase in N-cadherin expression, ultimately impacting the malignant progression of bladder tumors. SHMT2, as one of the serine hydroxymethyltransferase isozymes, is a potential cancer driver due to its influence on bladder cancer development, migration, and apoptosis.

### Supplementary Information

Supplementary material 1.

Supplementary material 2.

Supplementary material 3.

Supplementary material 4.

## Data Availability

The datasets used and/or analyzed during the current study available from the corresponding author on reasonable request.

## Accession code

BioProject accession number：PRJNA999182.
