# Supplementary material for: A study on the significance of serine hydroxymethyl transferase expression and its role in bladder cancer
Source: Sci Rep. 2024 Apr 9;14:8324. doi: 10.1038/s41598-024-58618-2 (PMC11003972; doi:10.1038/s41598-024-58618-2)
Supplement: Supplementary file 1 — Supplementary material 1. [file 41598_2024_58618_MOESM1_ESM.pdf]

Of all clinical specimens, 5 groups were used for transcriptome sequencing. Each group consisted of bladder cancer tissue and their adjacent non-tumor tissue. The following images are the basic results of transcriptome sequencing of these five groups of clinical samples. In the grouping label, Ca refers to bladder cancer tissue and CK refers to their adjacent non-tumor tissue. The specimens with the same numbers come from the same patient.

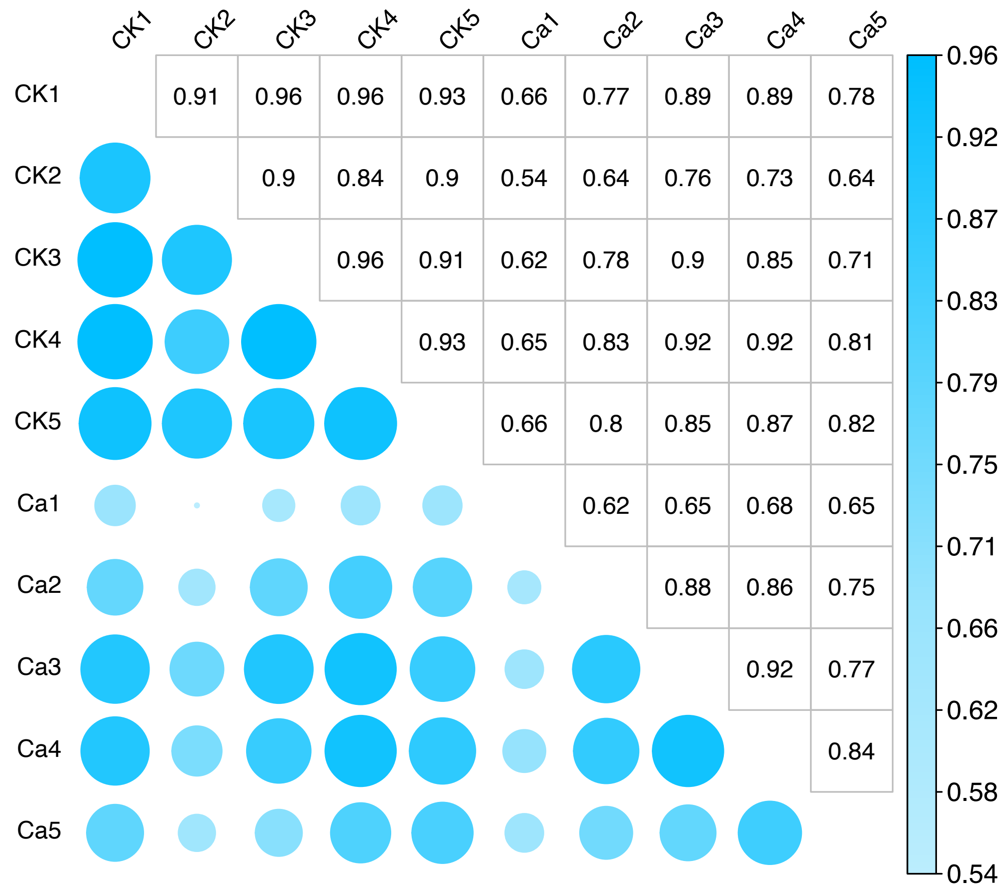

Figure 1. Correlation test between samples. According to the gene expression, the correlation coefficient diagram between sequencing samples is shown in the figure.

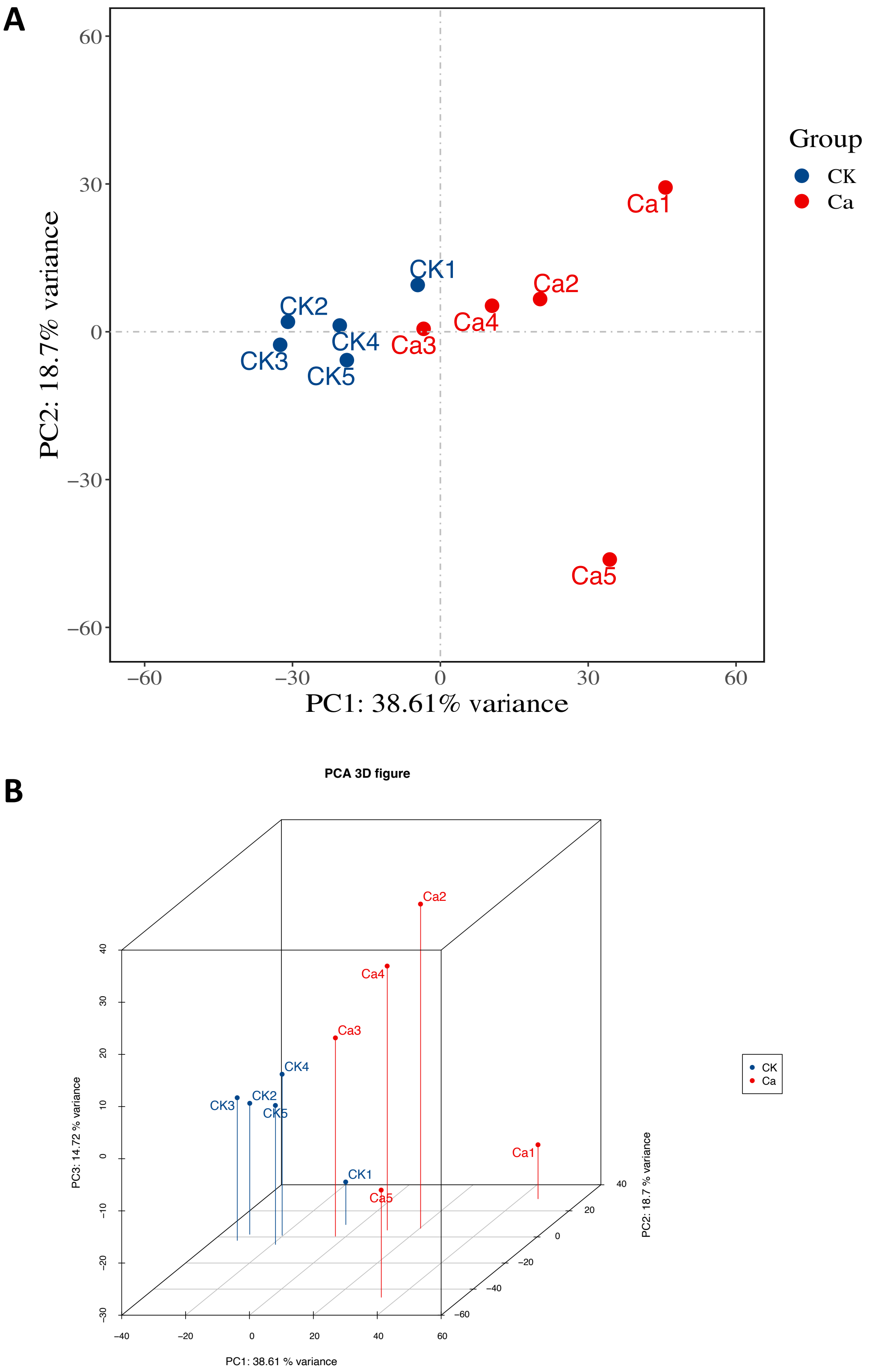

Figure 2. Principal component analysis between samples. (a) Two-dimensional principal component analysis. (b) Three-dimensional principal component analysis.

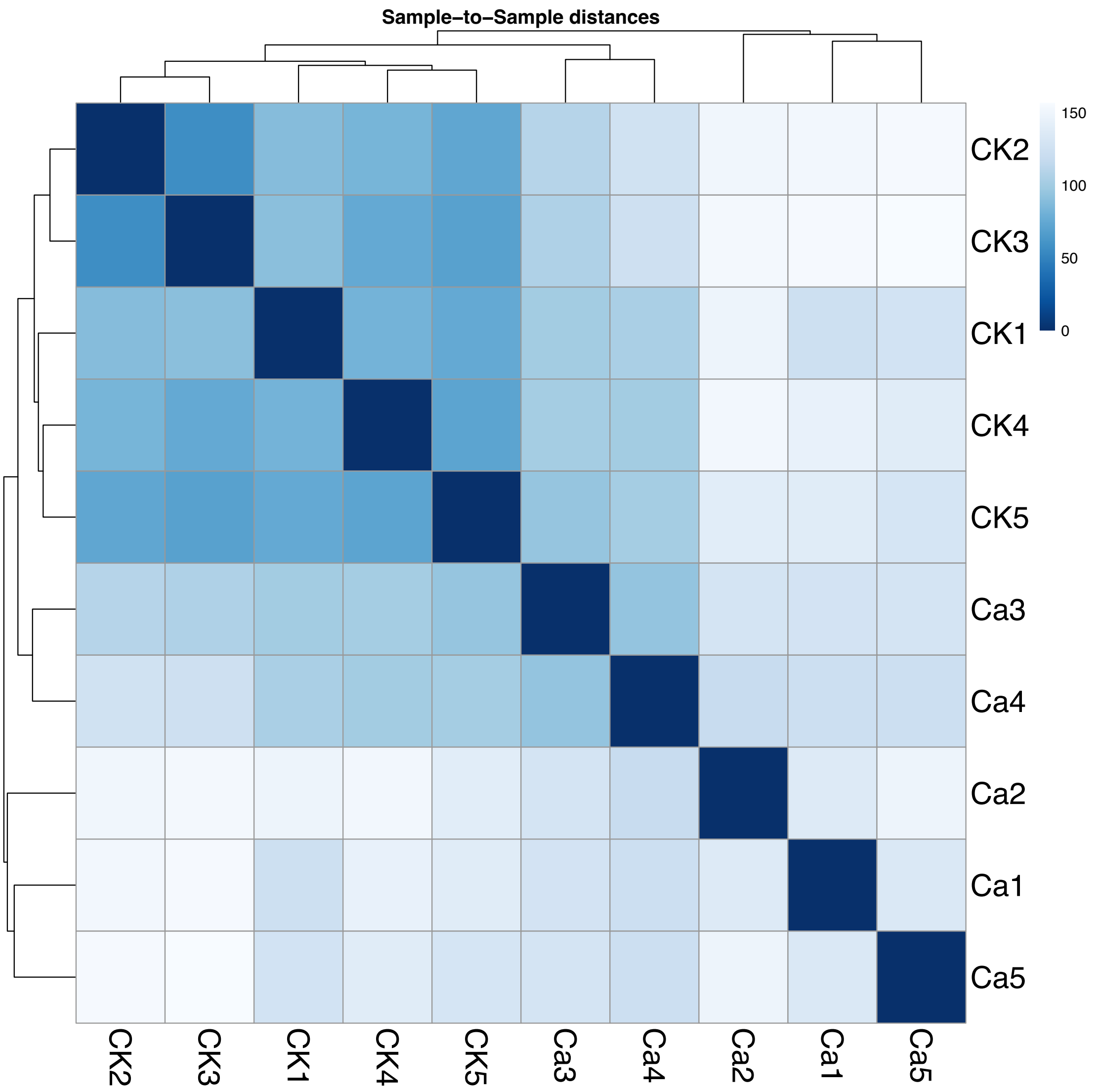

Figure 3. Sample-to-Sample cluster analysis. The cluster map of sequencing samples obtained according to gene expression is shown in the figure.

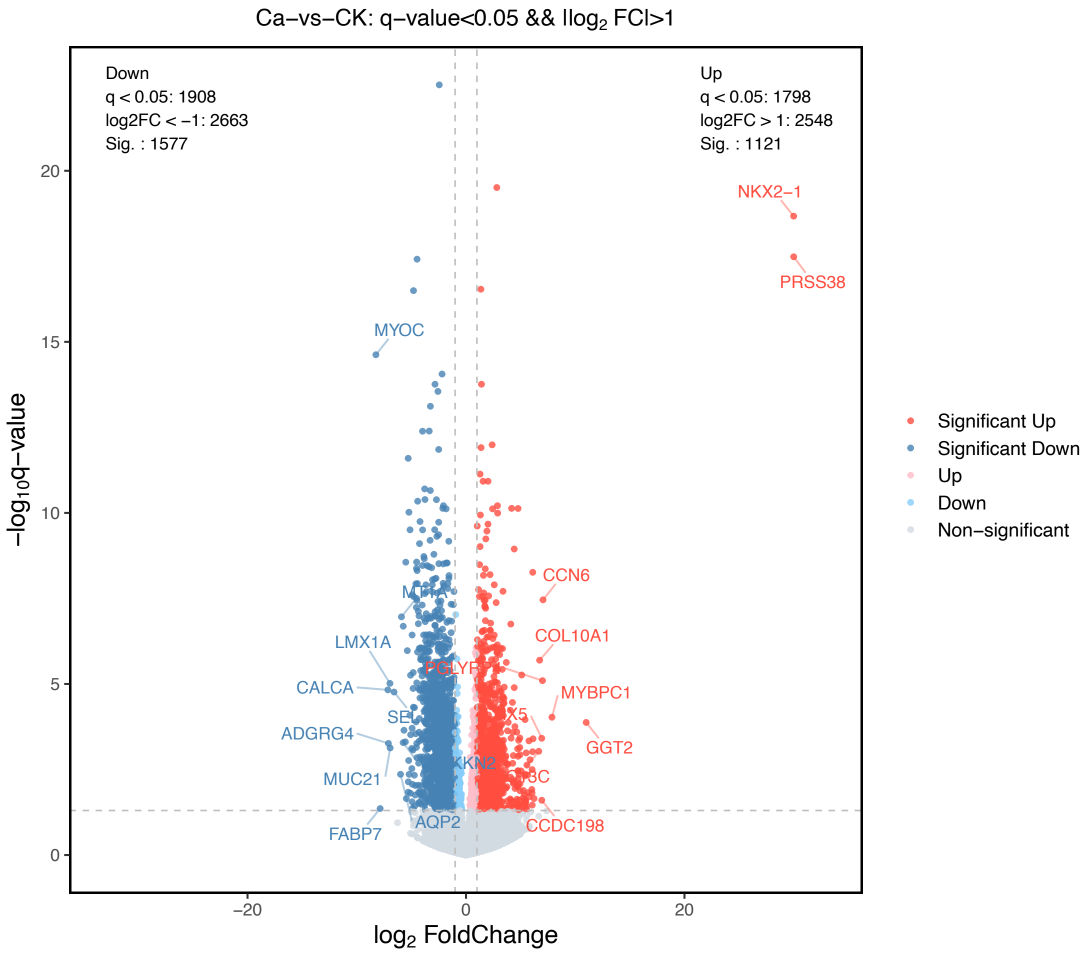

Figure 4. Volcano map of gene differential expression. The differential expression produced by the comparison is reflected in the volcanic map. The gray is the gene with no significant difference, while the red and blue are the genes with significant difference. The horizontal axis is log_2FoldChange, and the vertical axis is -log_{10}q-value.

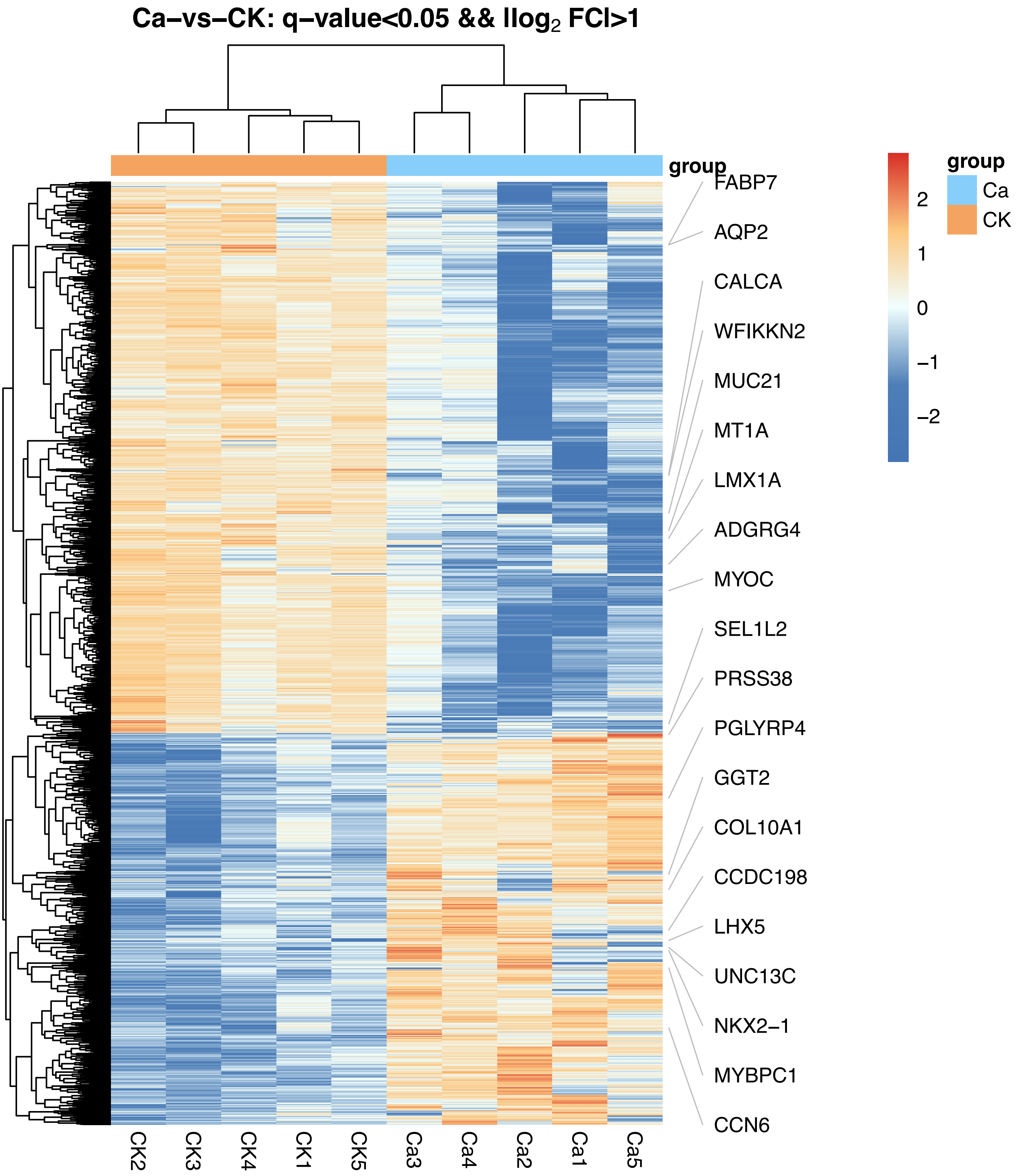

Figure 5. Differential gene grouping cluster map. Red represents a protein coding gene with relatively high expression, and blue represents a protein coding gene with relatively low expression.

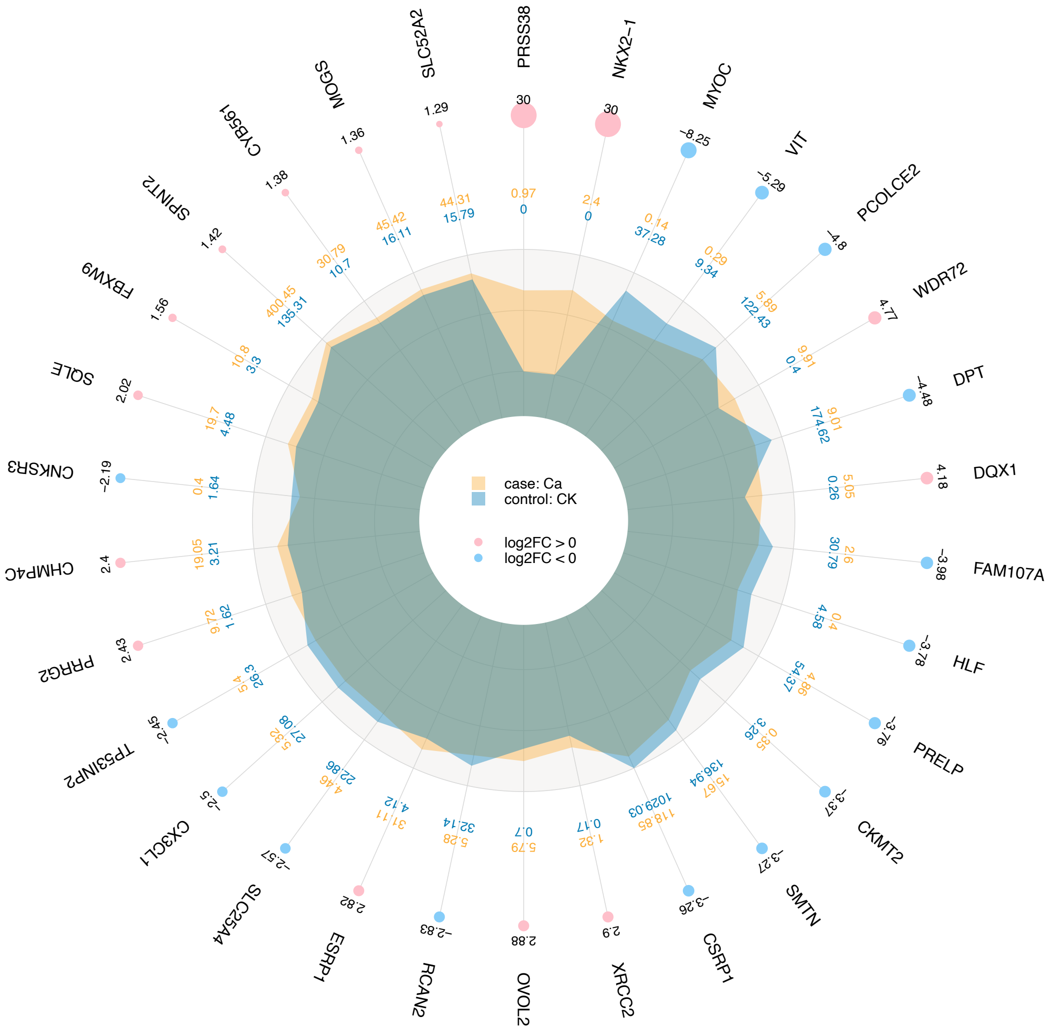

Figure 6. Radar map of differentially expressed genes. The first circle: Up-regulated gene (light red) and Down-regulated gene (light blue), and the size of the circle varies according to the value of log2(FC); The second circle: the outer circle data represents the average expression of the experimental group; The inner circle data represents the average expression of the control group; The third circle: the average expression of each gene in the experimental group and the control group. The radar map shows 30 up/down genes with the smallest Q value or P value.
