# Supplementary material for: A study on the significance of serine hydroxymethyl transferase expression and its role in bladder cancer
Source: Sci Rep. 2024 Apr 9;14:8324. doi: 10.1038/s41598-024-58618-2 (PMC11003972; doi:10.1038/s41598-024-58618-2)
Supplement: Supplementary file 2 — Supplementary material 2. [file 41598_2024_58618_MOESM2_ESM.pdf]

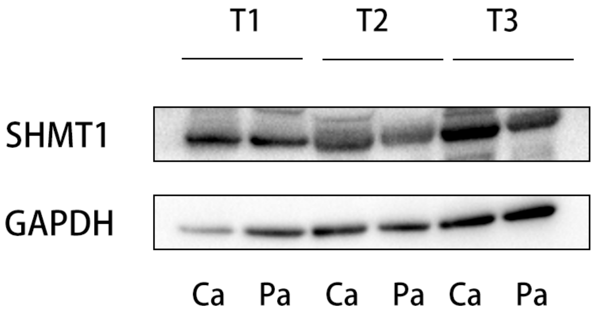

Supplementary material of Figure 4A in the manuscript

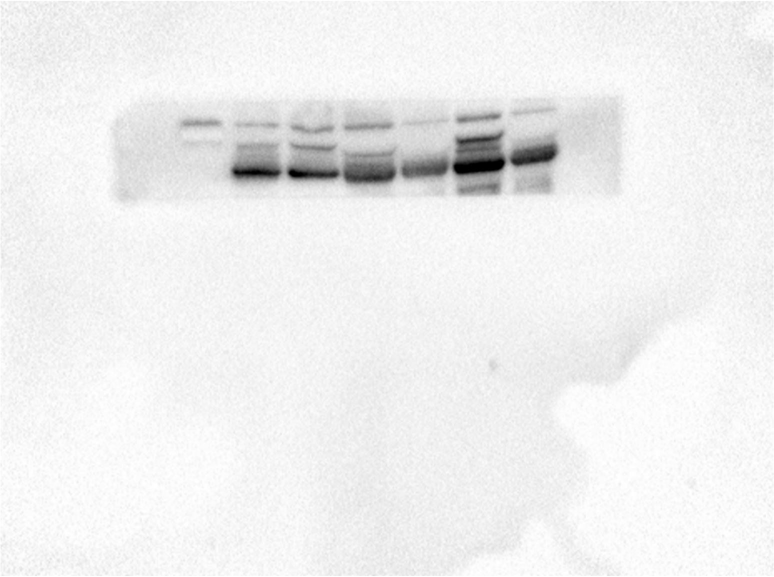

Clinical samples-SHMT1

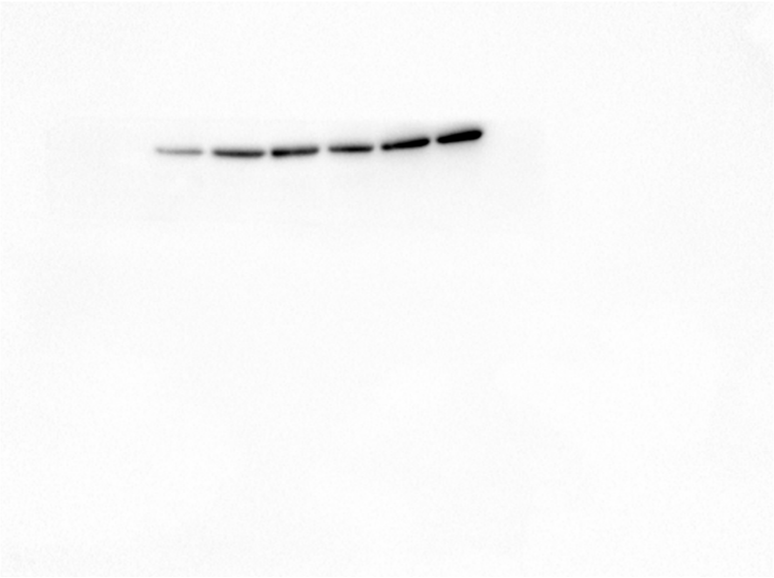

Clinical samples-GAPDH Reference to SHMT1

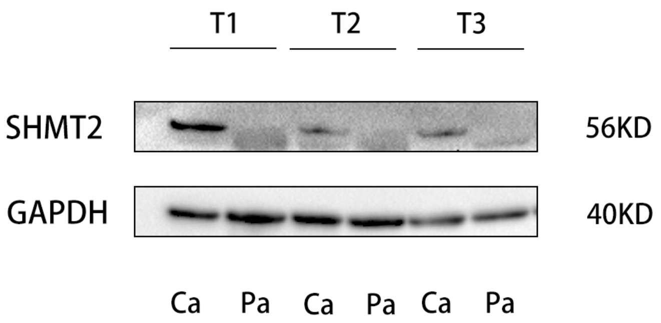

Supplementary material of Figure 4E in the manuscript

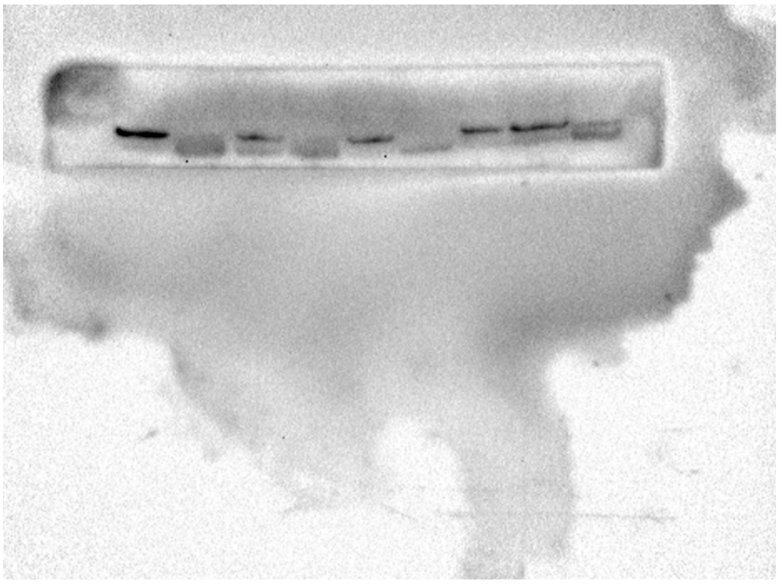

Clinical samples-SHMT2

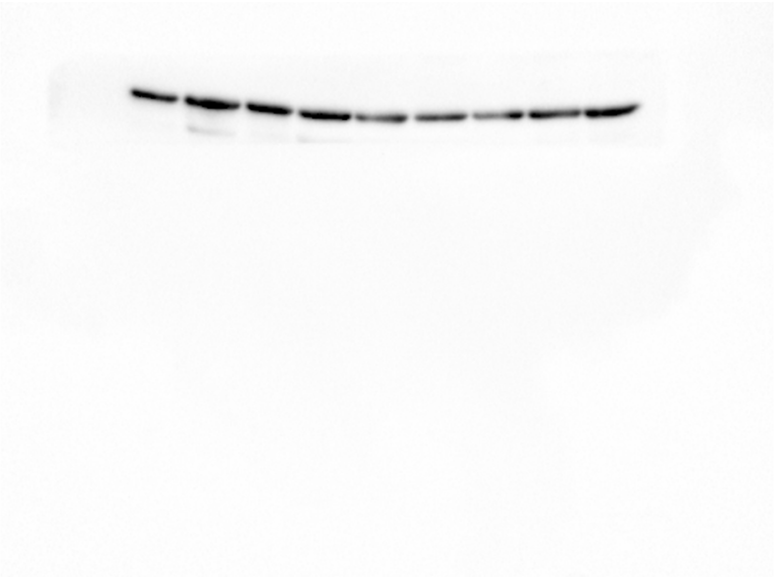

Clinical samples-GAPDH Reference to SHMT2
