# Supplementary material for: A study on the significance of serine hydroxymethyl transferase expression and its role in bladder cancer
Source: Sci Rep. 2024 Apr 9;14:8324. doi: 10.1038/s41598-024-58618-2 (PMC11003972; doi:10.1038/s41598-024-58618-2)
Supplement: Supplementary file 3 — Supplementary material 3. [file 41598_2024_58618_MOESM3_ESM.pdf]

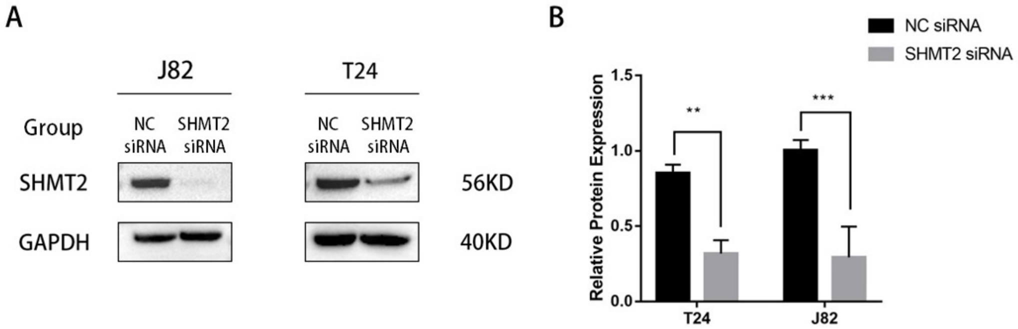

Supplementary material of Figure 6A in the manuscript

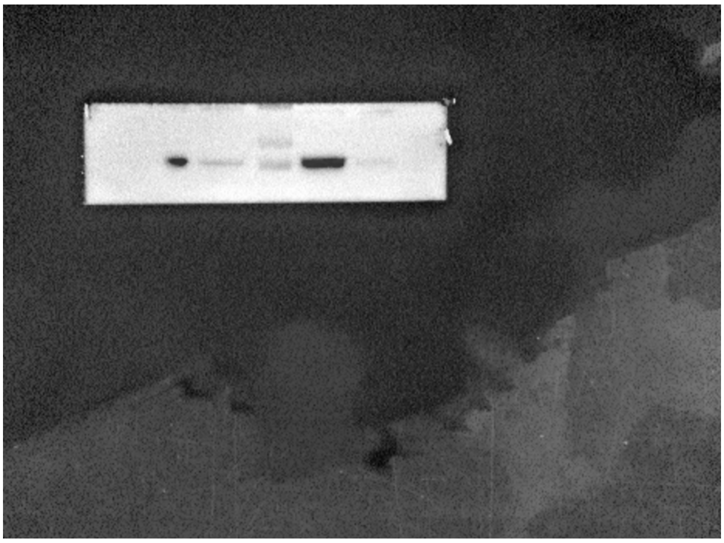

Protein expression of SHMT2, J82-SHMT2 control group and interference expression group, Biological repetition 1

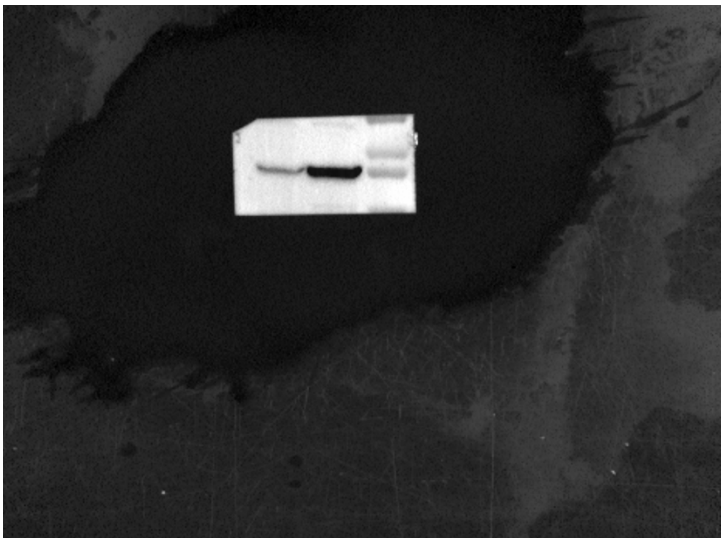

Protein expression of SHMT2, J82-SHMT2 interference expression group and control group, Biological repetition 2

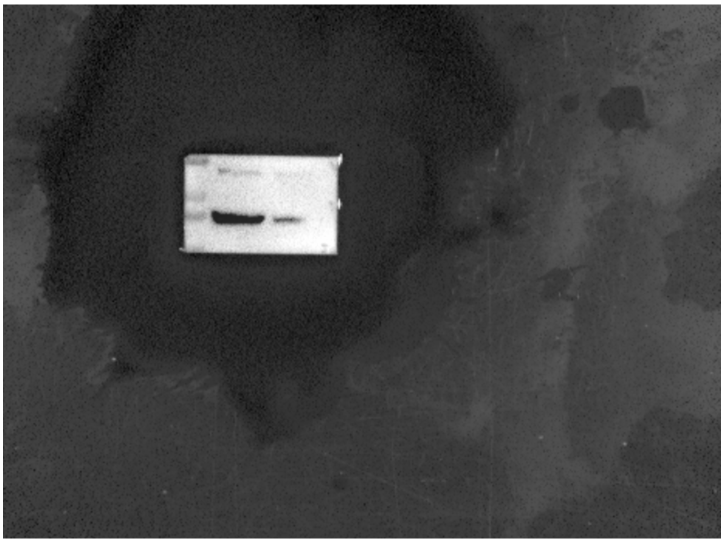

Protein expression of SHMT2, J82-SHMT2 control group and interference expression group, Biological repetition 3

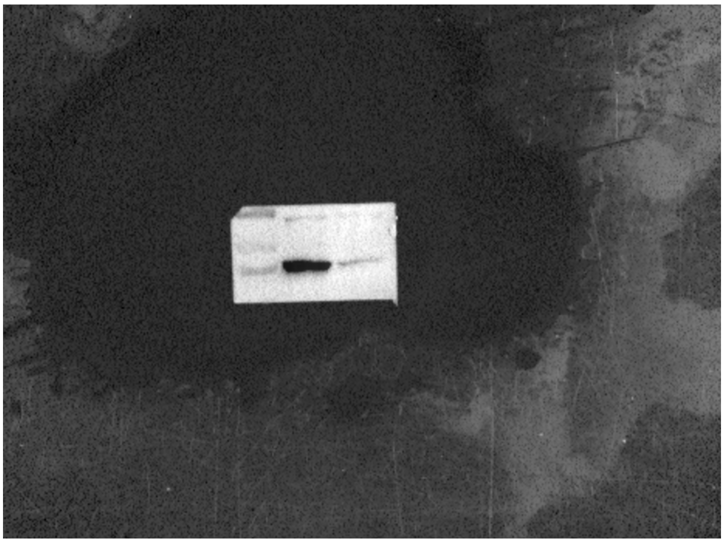

Protein expression of SHMT2, J82-SHMT2 control group and interference expression group, Biological repetition 4

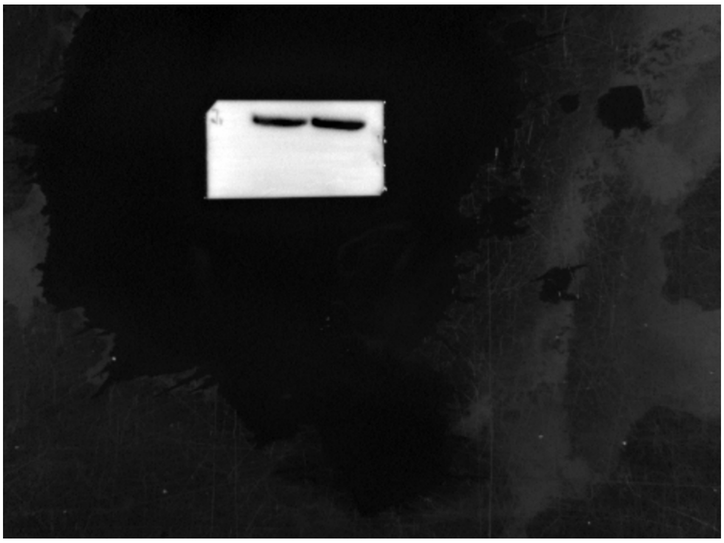

Protein expression of GAPDH, J82-SHMT2 control group and interference expression group

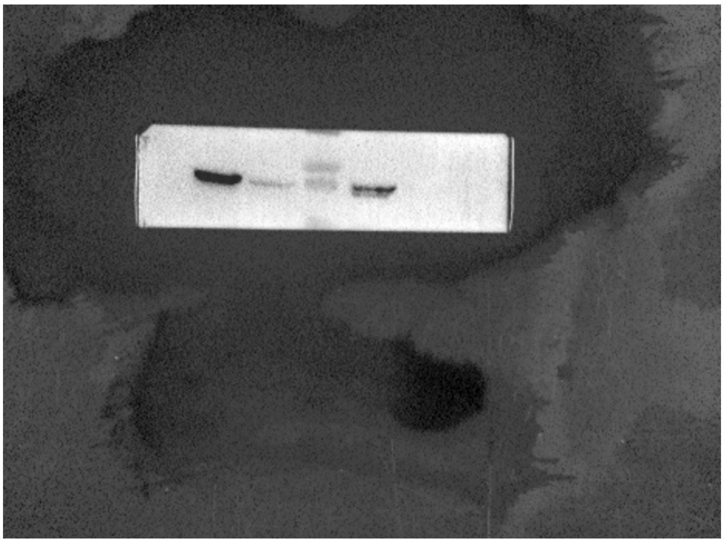

Protein expression of SHMT2, T24-SHMT2 control group and interference expression group, Biological repetition 1

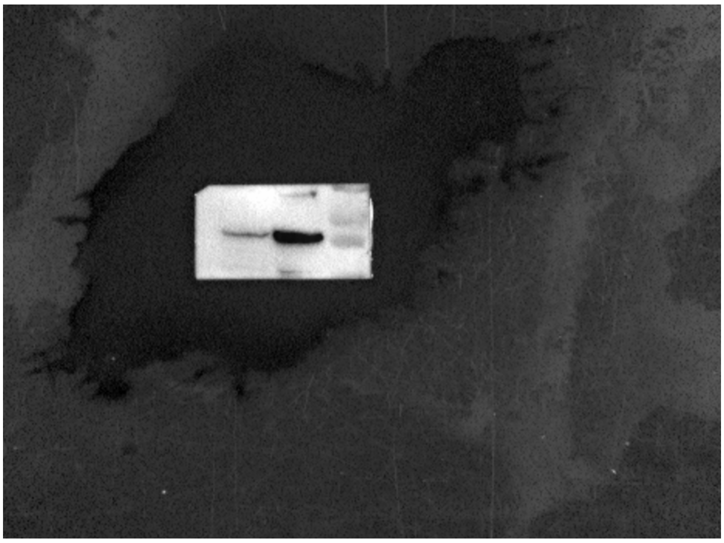

Protein expression of SHMT2, T24-SHMT2-2 interference expression group and control group, Biological repetition 2

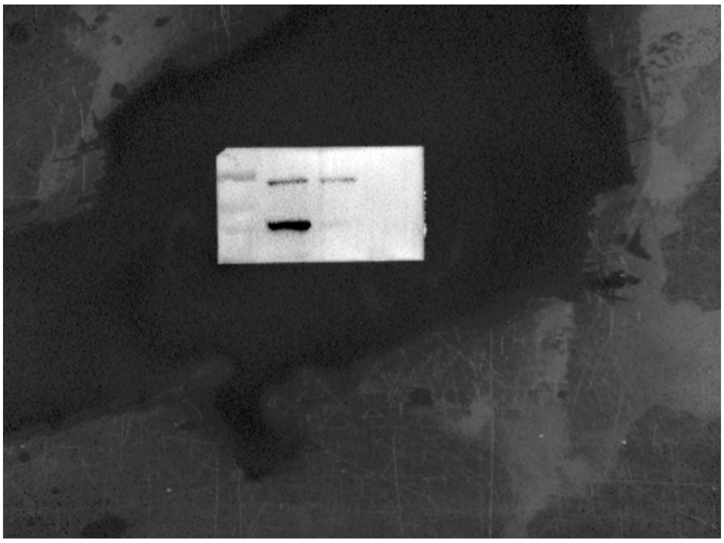

Protein expression of SHMT2, T24-SHMT2-3 control group and interference expression group,

Biological repetition 3

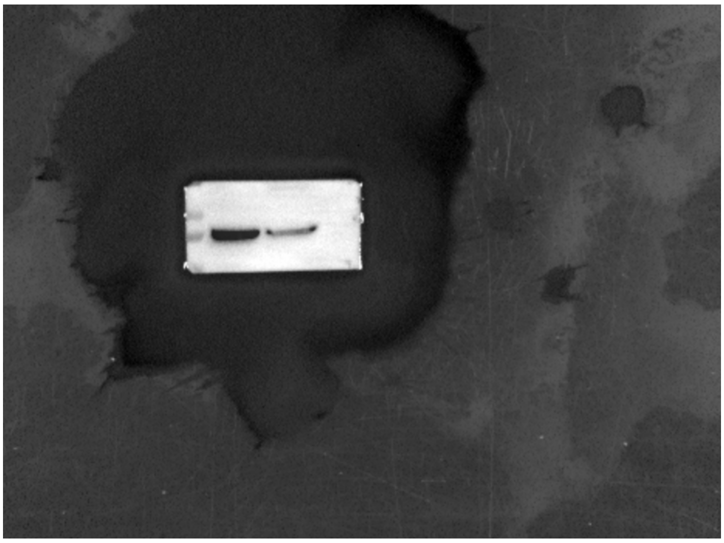

Protein expression of SHMT2, T24-SHMT2-4 control group and interference expression group,

Biological repetition 4

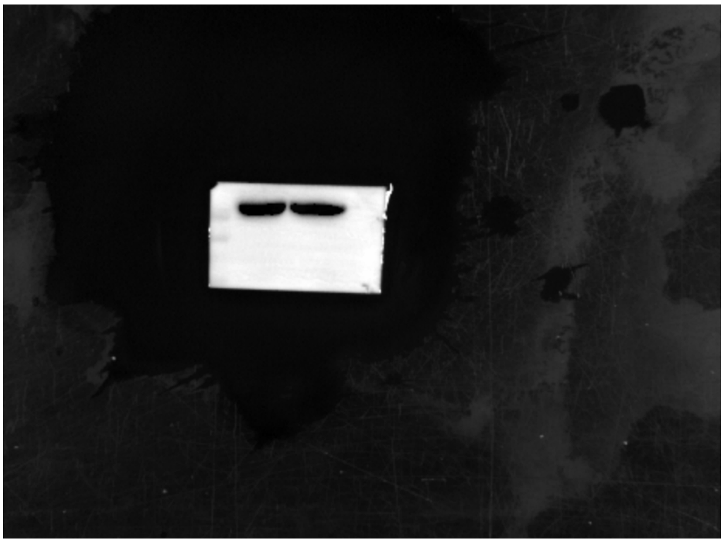

Protein expression of GAPDH, T24-SHMT2-4 control group and interference expression group
