# Supplementary material for: A study on the significance of serine hydroxymethyl transferase expression and its role in bladder cancer
Source: Sci Rep. 2024 Apr 9;14:8324. doi: 10.1038/s41598-024-58618-2 (PMC11003972; doi:10.1038/s41598-024-58618-2)
Supplement: Supplementary file 4 — Supplementary material 4. [file 41598_2024_58618_MOESM4_ESM.pdf]

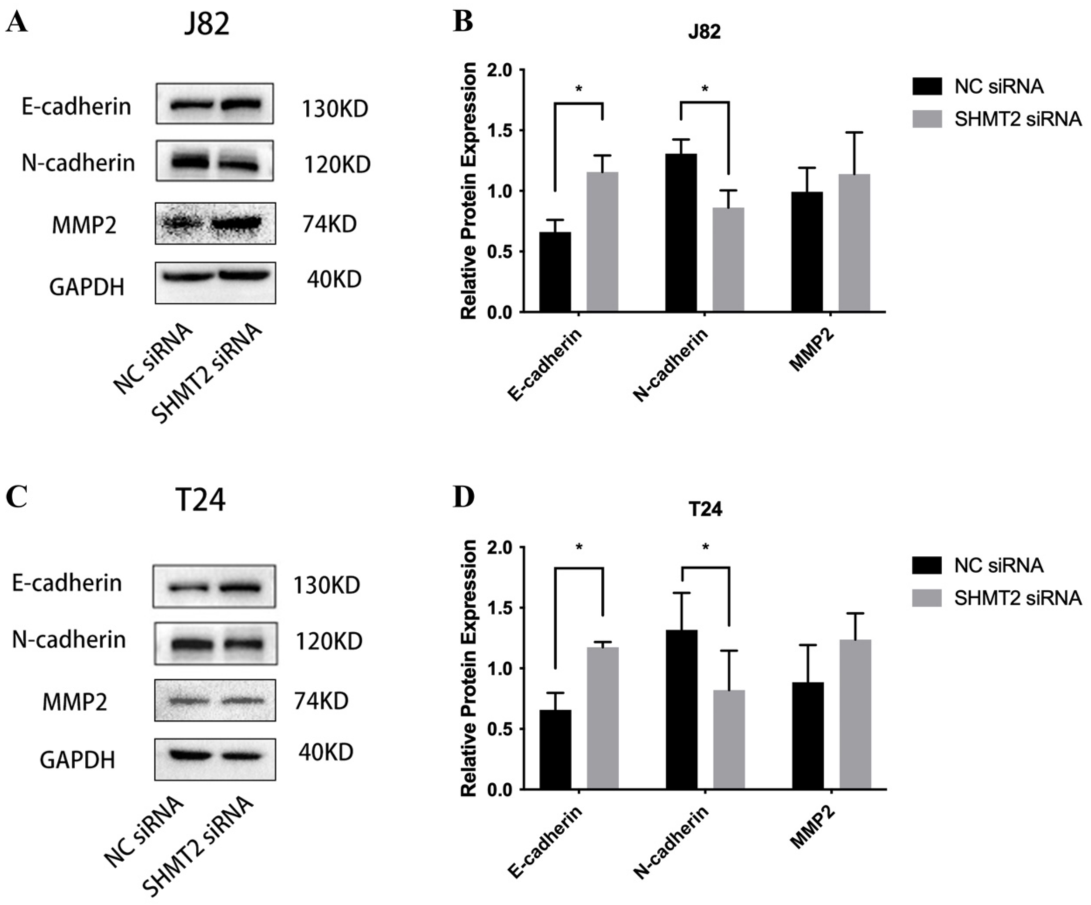

Supplementary material of Figure 7A and C in the manuscript

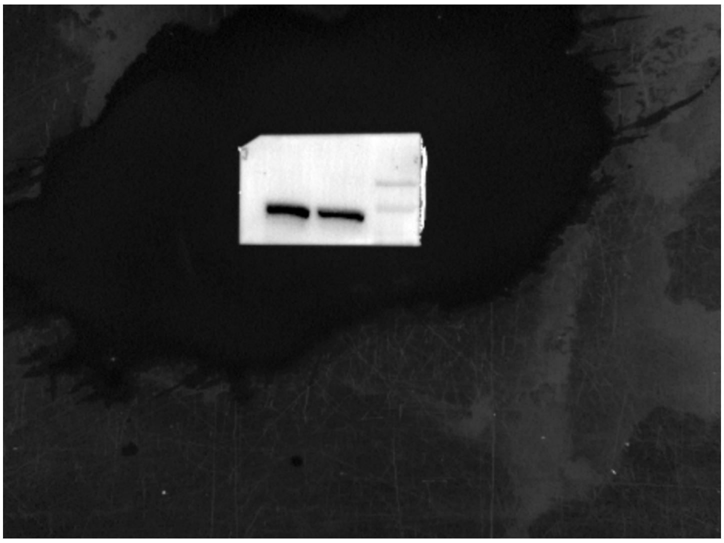

Protein expression of E-cadhering, J82-SHMT2 control group and interference expression group

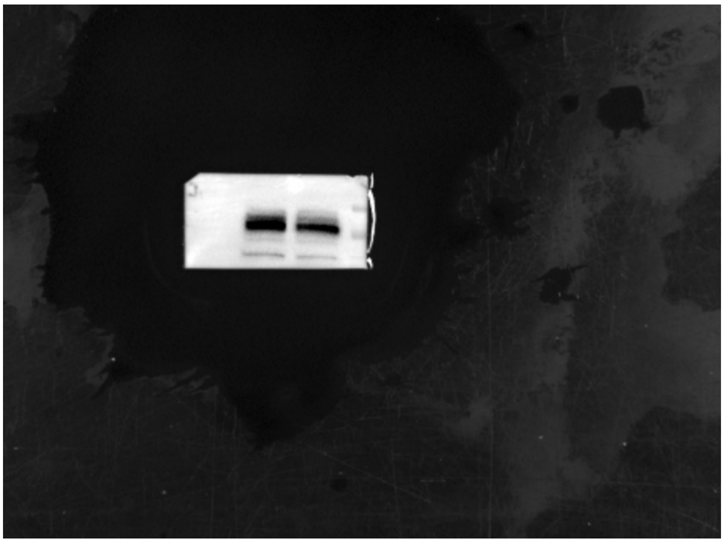
Protein expression of N-cadhering, J82-SHMT2 control group and interference expression group
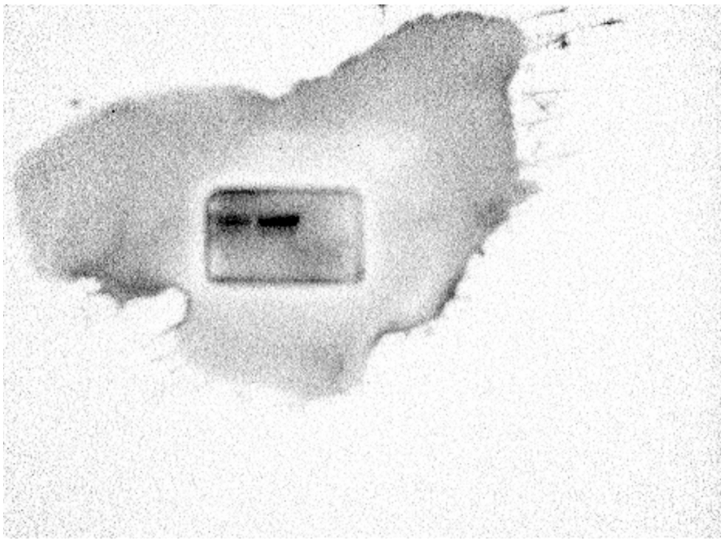
Protein expression of MMP2, J82-SHMT2 control group and interference expression group
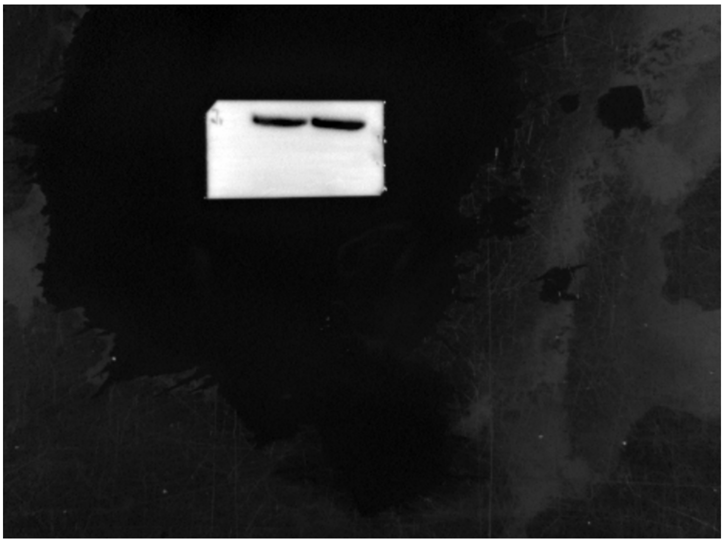
Protein expression of GAPDH, J82-SHMT2 control group and interference expression group

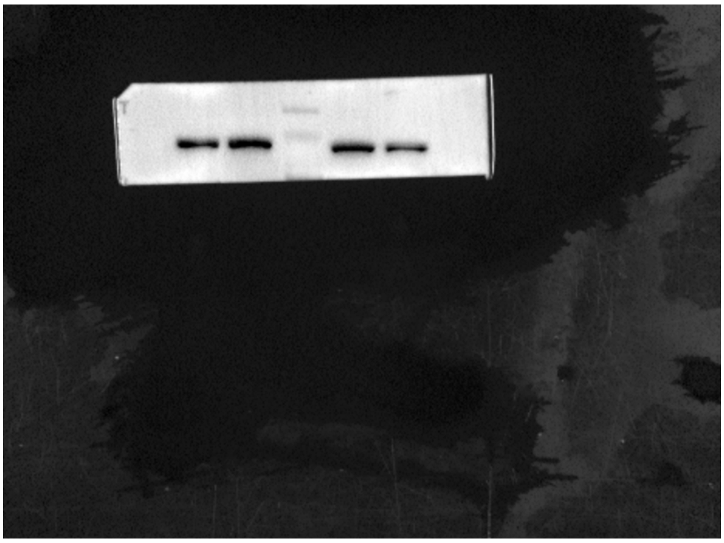
Protein expression of E-cadhering, T24-SHMT2 control group and interference expression group
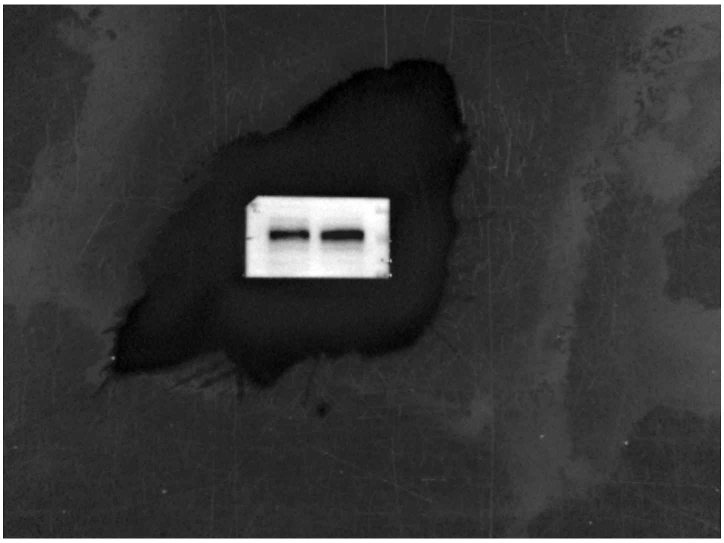
Protein expression of N-cadhering, T24-SHMT2 control group and interference expression group
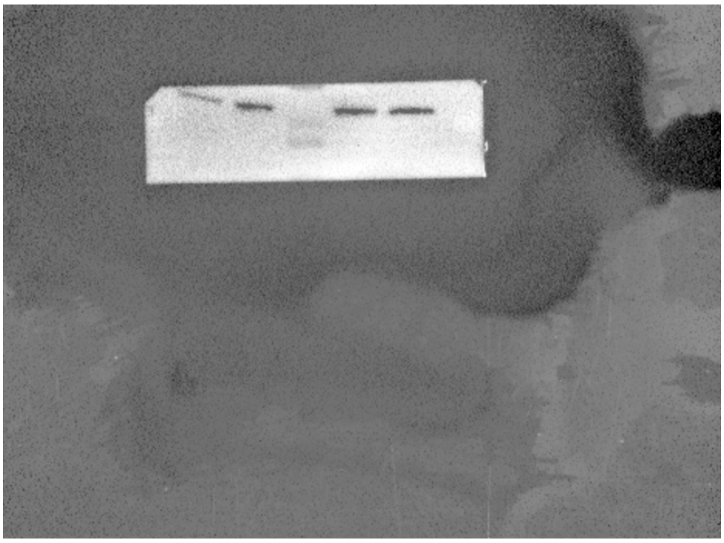
Protein expression of MMP2, T24-SHMT2 control group and interference expression group

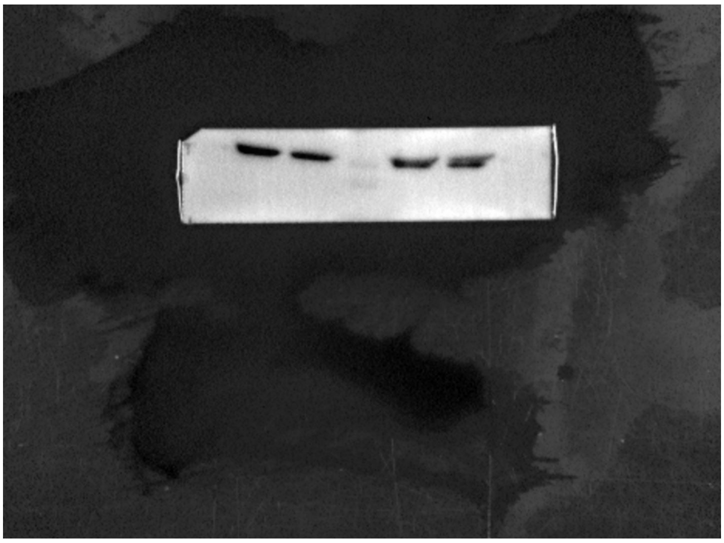

Protein expression of GAPDH, T24-SHMT2 control group and interference expression group
